# Global burden of lower respiratory infections attributable to secondhand smoke among children under 5 years of age, 2010–2019: a systematic analysis of the global burden of disease study 2019

**DOI:** 10.1186/s12889-023-16848-5

**Published:** 2023-10-04

**Authors:** Saina Xiang, Zhiyuan Chen, Zebin Dai, Fan Wang

**Affiliations:** 1 Medical Care Center, The First Affiliated Hospital of Wenzhou Medical University, Wenzhou, Zhejiang China; 2 Institute for Microbial Ecology, School of Medicine, Xiamen University, Xiamen, Fujian China; 3 Department of Gastroenterology, The Second Affiliated Hospital of Wenzhou Medical University, Wenzhou, Zhejiang China; 4 Department of Neurosurgery, The First Affiliated Hospital of Wenzhou Medical University, Wenzhou, Zhejiang China

**Keywords:** Global burden, Epidemiology, Child health, Lower respiratory infections, Secondhand smoke

## Abstract

**Background:**

Epidemiological trends of lower respiratory infections (LRIs) attributable to secondhand smoke (SHS) among children under 5 years since smoking bans have been increasingly applied globally remain unclear. Here, we aimed to estimate the spatiotemporal trends of the global, regional, and national burden of LRIs attributable to SHS among children under 5 years old between 2010 and 2019.

**Methods:**

Data on the deaths, and disability adjusted life years (DALYs) of the disease burden was retrieved from the Global Burden of Disease (GBD) 2019 for 204 countries and territories between 2010 and 2019. The rates per 100,000 population, along with 95% uncertainty intervals, as well as population-attributable fraction (PAF) was presented for each estimate.

**Results:**

In 2019, an estimated 6.94% (3.80–10.12%) of under-5 LRIs deaths were attributable to SHS globally, with an under-5 mortality rate of 7.02 per 100,000, a decrease of 5.77% since 2010. Similarly, 6.95% (3.81–10.13%) of LRIs DALYs were due to SHS among children under 5 years, with a rate in under-5s of 619.36 DALYs per 100,000, and also a 5.77% decrease since 2010. Azerbaijan, Turkmenistan, and Papua New Guinea showed the highest under-5 mortality and DALYs burden rates of LRIs attributable to SHS in 2019. In contrast, the PAF was stagnant over the past ten years and there is even a year-on-year upward trend in South Asia. Nationally, in 2019, Bosnia and Herzegovina, Armenia, and Montenegro showed the highest PAF_SHS_ of LRIs burden among children under 5 years of age. In addition, the burden was heavier in children under 1 year of age and was significantly negatively associated with sociodemographic index.

**Conclusions:**

SHS remains a risk factor that cannot be ignored for LRIs burden worldwide. Hence, governments and health systems should continue to take steps to reduce SHS pollution among young children to mitigate this burden.

**Supplementary Information:**

The online version contains supplementary material available at 10.1186/s12889-023-16848-5.

## Introduction

Lower respiratory infections (LRIs), mainly caused by bacteria such as Streptococcus pneumoniae and Haemophilus influenzae type b and viruses such as influenza and respiratory syncytial virus [1, 2], are a leading cause of death globally, killing more than 2 million people every year [3]. In particular, LRIs are a substantial cause of deaths among children under 5 years of age [4, 5] and approximately 0.67 million children under 5 years died from LRIs globally in 2019 [6]. Despite large reductions in under-5 LRIs mortality in many locations since the 1990s, the pace of progress for LRIs has generally lagged behind that of other childhood infectious diseases [7].

Secondhand smoke (SHS) exposure has been highlighted as a significant contributor to the burden of deaths from respiratory diseases [8, 9]. Children are particularly vulnerable to the harmful health effects of SHS as their immune and respiratory systems are less developed and they have a faster respiratory rate [10]. In addition, as young children like to be held or sit on their parents’ laps, they are often very close to sources of SHS [11].

Several analytical epidemiological studies have reported the burden of respiratory disease attributable to SHS exposure for many sites worldwide, including whole countries [12–14], and these provide real burden data. The most recent study based on the worldwide burden of LRIs attribute to SHS was from 192 countries during 2004, which showed that 30% of all deaths from SHS occur in children, with the largest disease burden from LRIs in those younger than 5 years of age [9]; these estimates need updating and the trends followed by years has not been reported. In addition, it was reported that only 7.4% of the world population lives in jurisdictions with comprehensive smoke-free laws in 2009, and the enforcement of these laws is robust in only a few of those jurisdictions [15]. With the recommendation of the World Health Organization (WHO) Framework Convention on Tobacco Control [16], updated information on the burden of LRIs due to SHS is needed for public health and advocacy purposes. Whatmore, it was reported that children under 5 years of age living in deprived communities were more likely to be exposed to SHS in the home [17]. Assessment of the impact of socioeconomic status on the burden of disease from SHS is also desired.

Thus, based on the latest data and improved methodologies of the Global Burden of Disease (GBD) 2019, we aimed to estimate the spatiotemporal trends of SHS-related LRIs burden and identified the highly affected regions, as well as the influence derived by the sociodemographic index (SDI) in 204 countries and territories from 2010 to 2019, providing insight to assist policymaking and recommending actions to reduce SHS exposure and its corresponding LRIs burden.

## Methods

### Data source

Original data sources on the global burden of LRIs attributable to SHS were obtained from the GBD 2019 (https://ghdx.healthdata.org/). GBD studies provide worldwide and comprehensive assessments of health loss for 329 diseases across 204 countries and territories that are classified into 21 regions according to epidemiological similarities and geographical proximity [3]. Detailed descriptions of the methodologies have been reported elsewhere [18, 19] and fatal and non-fatal estimates have been published (https://vizhub.healthdata.org/gbd-compare/ and https://ghdx.healthdata.org/gbd-results-tool).

### Estimation of SHS and its attributable LRIs Burden

In GBD 2019, LRIs were defined as clinician-diagnosed pneumonia or bronchiolitis, with International Classification of Diseases (ICD) 9th edition coded as 073.0-073.6, 079.82, 466–469, 480–489, 513.0, and 770.0, and ICD 10th edition coded as A48.1, J09-J22, J85.1, P23-P23.9, and U04. SHS refers to current exposure to secondhand tobacco smoke at home, at work, or in other public places [18].

The LRIs burden of SHS was estimated by calculating the attributable composition in death and disability-adjusted life years (DALYs). The methods on the estimations of LRIs attributable to SHS have been introduced in detail previously [18]. In brief, the GBD 2019 estimation of attributable burden followed the general framework established for comparative risk assessment, which can be divided into six key steps: identifying the SHS-LRIs pair in the analysis; estimating relative risk as a function of exposure (SHS); estimating the distribution of SHS exposure levels by age-sex-location-year; determining the theoretical minimum risk exposure level (TMREL), which is the level of risk exposure (SHS) that minimises risk at the population level, or the level of risk that captures the maximum attributable burden; and estimating the population attributable fractions and attributable burden by age-sex-location-year using a formula taking into account the risk function, the distribution of exposure across individuals in each age-sex-location-year, and the TMREL [18].

DALYs is a commonly used comprehensive indicator that assesses the overall impact of disease and injury on a population by combining years of healthy life lost due to premature death (YLL) and years lived with a disability (YLD). YLL calculates potential years of life lost by subtracting age at death from life expectancy, while YLD considers years lived with a disability, using a disability weight reflecting the condition’s severity. Higher DALYs values signify greater health impact, enabling effective cross-condition, regional, and temporal comparisons to inform public health strategies and gauge intervention success. The level of uncertainty was calculated by sampling 1000 draws at each computational step and combining uncertainty from several different sources (that is, input data, corrections of measurement error, and estimates of residual non-sampling error). The uncertainty intervals were defined as the 25th and 975th values of the ordered draws.

The relation between the burden of LRIs and the SDI for the 21 regions and 204 countries and territories was examined with smoothing spline models [20]. The SDI is a composite measure that quantifies the development level of a country or region based on its socioeconomic indicators. It takes into account three key dimensions: income per capita and consists of the gross domestic product per capita (smoothed over the previous decade), average number of years of education for the population (> 15 years old), and total fertility rate in those aged < 25 years. The scoring mechanism of the SDI involves transforming each component’s value into a normalized score between 0 and 1. The normalized values are then geometrically averaged to obtain the final SDI score for a country or region. The resulting SDI score ranges from 0 (lowest development) to 1 (highest development). This index enables comparisons of health outcomes, such as disease burden and life expectancy, across different countries or regions with similar levels of development.

### Statistical analyses

We present estimates (with 95% uncertainty intervals [UIs]) of population-attributable fractions (PAF), rates and estimated the annual percentage change (EAPC) per 100,000 population of LRIs deaths and DALYs attributable to SHS. The PAF is determined by comparing the health outcomes (e.g., deaths) in the presence of SHS to the health outcomes that would be observed if that SHS were absent, while accounting for other relevant factors. Here’s the general formula for calculating PAF: PAF=(RR–1)/RR, where RR is the relative risk. A higher PAF indicates that a greater portion of the LRIs burden can be attributed to SHS, emphasizing the potential benefit of targeting SHS to reduce the overall burden of the LRIs. EAPC is a summary and widely used measure of the trend of rate over a specific time interval and is calculated with linear regression models as follows: ln (rate) = α + β*calendar year + ε. The EAPC was defined as 100 × (exp (β) − 1), and its 95% UIs were determined by the regression model. Statistical analysis was performed using R (version 4.1.0). *P* value < 0.05 was considered statistically significant.

## Results

### Global level

In 2019, 6.94% (3.80–10.12%) of under-5 LRIs deaths were attributable to SHS globally, with an under-5 mortality rate of 7.02 per 100,000, a decrease of 5.77% since 2010. In addition, 6.95% (3.81–10.13%) of under-5 LRIs DALYs were due to SHS, with a rate in under-5s of 619.36 DALYs per 100,000, a 5.77% decrease since 2010 (Table 1).

**Table 1:** Global burden of LRIs attributable to SHS among children under 5 years of age*

	Deaths						DALYs				
	2010		2019		2010–2019		2010		2019		2010–2019
	Percentage, %	Rate, 10^5^	Percentage, %	Rate, 10^5^	EAPC, %		Percentage, %	Rate, 10^5^	Percentage, %	Rate, 10^5^	EAPC, %
Global	7.41 (4.07 to 10.92)	11.91 (6.54 to 18.12)	6.94 (3.80 to 10.12)	7.02 (3.75 to 10.83)	-5.77 (-6.18 to -5.36)		7.42 (4.07 to 10.93)	1,050.08 (577.08 to 1,594.57)	6.95 (3.81 to 10.13)	619.36 (330.98 to 955.38)	-5.77 (-6.17 to -5.36)
Andean Latin America	2.77 (0.99 to 5.19)	2.91 (1.02 to 5.53)	2.86 (1.05 to 5.33)	1.50 (0.48 to 2.89)	-6.93 (-7.32 to -6.54)		2.77 (0.99 to 5.19)	256.21 (90.07 to 487.27)	2.86 (1.05 to 5.33)	132.30 (42.57 to 254.79)	-6.92 (-7.30 to -6.53)
Australasia	12.89 (7.65 to 18.31)	0.36 (0.21 to 0.53)	12.19 (7.34 to 17.66)	0.24 (0.13 to 0.36)	-4.33 (-4.73 to -3.94)		12.89 (7.66 to 18.32)	32.72 (19.28 to 48.25)	12.19 (7.34 to 17.66)	21.55 (12.16 to 32.67)	-4.29 (-4.66 to -3.91)
Caribbean	3.41 (1.66 to 5.54)	4.20 (1.93 to 7.10)	3.63 (1.80 to 5.79)	3.44 (1.57 to 5.92)	-2.29 (-2.70 to -1.87)		3.41 (1.66 to 5.54)	370.30 (170.28 to 624.46)	3.64 (1.80 to 5.79)	303.10 (138.49 to 521.90)	-2.29 (-2.70 to -1.88)
Central Asia	13.81 (8.75 to 19.01)	30.80 (19.16 to 43.09)	13.22 (8.49 to 18.22)	16.60 (9.96 to 24.04)	-6.72 (-6.93 to -6.51)		13.81 (8.75 to 19.01)	2,712.69 (1,687.90 to 3,795.10)	13.22 (8.50 to 18.22)	1,461.52 (876.67 to 2,116.69)	-6.72 (-6.93 to -6.51)
Central Europe	19.42 (12.65 to 26.10)	3.87 (2.49 to 5.19)	18.16 (11.50 to 24.57)	2.07 (1.25 to 2.98)	-6.37 (-6.77 to -5.96)		19.41 (12.65 to 26.09)	342.54 (220.52 to 459.20)	18.16 (11.50 to 24.57)	184.17 (111.31 to 264.58)	-6.34 (-6.75 to -5.93)
Central Latin America	4.11 (1.64 to 7.35)	2.25 (0.85 to 4.04)	4.02 (1.63 to 7.08)	1.42 (0.52 to 2.63)	-5.24 (-5.48 to -5.00)		4.11 (1.64 to 7.35)	198.28 (74.93 to 356.58)	4.02 (1.63 to 7.08)	125.08 (46.14 to 232.34)	-5.23 (-5.47 to -4.99)
Central Sub-Saharan Africa	3.13 (1.43 to 5.15)	8.05 (3.66 to 13.79)	3.10 (1.40 to 5.15)	3.47 (1.49 to 6.10)	-9.07 (-9.33 to -8.80)		3.13 (1.43 to 5.15)	706.95 (321.82 to 1,211.38)	3.10 (1.39 to 5.14)	305.80 (131.06 to 537.04)	-9.05 (-9.31 to -8.78)
East Asia	19.71 (13.10 to 26.45)	9.26 (5.94 to 12.39)	19.59 (13.03 to 26.35)	3.57 (2.27 to 4.93)	-9.72 (-10.55 to -8.88)		19.71 (13.10 to 26.45)	818.06 (525.00 to 1,094.79)	19.59 (13.03 to 26.35)	316.41 (201.13 to 437.08)	-9.70 (-10.53 to -8.86)
Eastern Europe	20.92 (13.79 to 27.94)	3.85 (2.48 to 5.18)	20.78 (13.72 to 27.61)	2.25 (1.42 to 3.15)	-7.44 (-9.32 to -5.52)		20.92 (13.80 to 27.94)	340.66 (219.80 to 457.63)	20.78 (13.72 to 27.61)	199.88 (126.17 to 279.10)	-7.40 (-9.27 to -5.49)
Eastern Sub-Saharan Africa	3.31 (1.31 to 5.86)	8.66 (3.23 to 15.78)	3.60 (1.51 to 6.22)	5.41 (2.11 to 10.10)	-5.12 (-5.45 to -4.79)		3.31 (1.31 to 5.87)	761.18 (284.19 to 1,386.58)	3.60 (1.50 to 6.22)	475.59 (185.38 to 887.42)	-5.12 (-5.44 to -4.79)
High-income Asia Pacific	17.47 (11.36 to 23.84)	0.57 (0.37 to 0.78)	16.88 (10.89 to 23.07)	0.35 (0.23 to 0.50)	-5.44 (-5.70 to -5.18)		17.47 (11.36 to 23.84)	51.18 (32.97 to 69.80)	16.88 (10.89 to 23.07)	32.04 (20.34 to 44.67)	-5.33 (-5.58 to -5.08)
High-income North America	12.67 (8.16 to 17.37)	0.40 (0.26 to 0.55)	12.82 (8.33 to 17.90)	0.35 (0.22 to 0.49)	-1.25 (-1.56 to -0.94)		12.67 (8.16 to 17.37)	35.82 (23.24 to 49.49)	12.81 (8.32 to 17.89)	31.45 (20.41 to 44.09)	-1.22 (-1.52 to -0.91)
North Africa and Middle East	13.47 (8.55 to 18.51)	12.39 (7.61 to 17.71)	12.95 (8.23 to 17.93)	6.65 (3.87 to 9.75)	-6.73 (-7.12 to -6.34)		13.48 (8.55 to 18.51)	1,092.24 (671.47 to 1,561.35)	12.95 (8.23 to 17.93)	586.61 (341.88 to 860.85)	-6.72 (-7.11 to -6.34)
Oceania	9.69 (4.55 to 15.60)	35.79 (15.21 to 62.13)	9.72 (4.62 to 15.56)	27.53 (11.95 to 46.36)	-2.85 (-2.96 to -2.74)		9.69 (4.55 to 15.59)	3,147.01 (1,337.22 to 5,465.07)	9.72 (4.62 to 15.56)	2,421.89 (1,050.58 to 4,081.08)	-2.84 (-2.95 to -2.73)
South Asia	9.28 (5.03 to 13.80)	18.66 (9.96 to 28.46)	9.64 (5.30 to 14.19)	11.18 (5.91 to 17.19)	-5.81 (-6.48 to -5.13)		9.29 (5.03 to 13.81)	1,645.67 (878.54 to 2,511.16)	9.64 (5.30 to 14.19)	987.25 (521.73 to 1,516.93)	-5.79 (-6.46 to -5.12)
Southeast Asia	12.97 (7.25 to 18.80)	13.79 (7.47 to 20.99)	13.37 (7.68 to 19.46)	8.20 (4.43 to 12.34)	-5.52 (-5.69 to -5.36)		12.97 (7.24 to 18.80)	1216.34 (659.34 to 1852.95)	13.37 (7.68 to 19.46)	722.94 (391.05 to 1,089.19)	-5.52 (-5.69 to -5.36)
Southern Latin America	14.98 (8.98 to 21.21)	2.66 (1.58 to 3.77)	14.48 (8.59 to 20.76)	1.59 (0.85 to 2.45)	-5.80 (-6.38 to -5.21)		14.97 (8.98 to 21.20)	235.64 (139.91 to 334.73)	14.48 (8.59 to 20.76)	140.83 (75.70 to 217.12)	-5.78 (-6.37 to -5.20)
Southern Sub-Saharan Africa	7.76 (3.98 to 11.78)	13.05 (6.53 to 20.40)	7.50 (3.92 to 11.42)	9.12 (4.40 to 14.82)	-4.11 (-4.33 to -3.88)		7.76 (3.98 to 11.78)	1,153.19 (577.71 to 1,804.18)	7.50 (3.92 to 11.42)	804.56 (388.10 to 1,306.96)	-4.13 (-4.35 to -3.90)
Tropical Latin America	9.80 (5.65 to 14.44)	5.57 (3.12 to 8.49)	10.55 (6.49 to 15.03)	3.29 (1.85 to 4.89)	-6.03 (-6.57 to -5.49)		9.80 (5.65 to 14.44)	491.22 (275.69 to 748.46)	10.55 (6.49 to 15.04)	290.02 (162.93 to 431.85)	-6.02 (-6.56 to -5.48)
Western Europe	14.95 (9.33 to 20.56)	0.27 (0.17 to 0.38)	14.44 (8.99 to 19.99)	0.19 (0.11 to 0.27)	-3.76 (-4.30 to -3.21)		14.94 (9.33 to 20.57)	25.29 (15.68 to 35.09)	14.44 (8.99 to 19.98)	17.55 (10.81 to 25.30)	-3.63 (-4.16 to -3.09)
Western Sub-Saharan Africa	3.38 (1.68 to 5.25)	14.50 (6.99 to 24.00)	3.47 (1.80 to 5.37)	11.04 (5.31 to 18.30)	-2.6 (-3.15 to -2.04)		3.38 (1.68 to 5.25)	1,271.73 (613.12 to 2,105.47)	3.47 (1.80 to 5.37)	968.43 (467.10 to 1,604.96)	-2.59 (-3.15 to -2.04)

* LRIs deaths and DALYs attribute to SHS among children under 5 years of age in 21 regions and globally, and change from 2010 to 2019

LRIs: lower respiratory infections; SHS: secondhand smoke; EAPC: estimated the annual percentage change; DALYs: disability-adjusted life-years

### Regional level

In 2019, Oceania, Central Asia, and South Asia had the highest under-5 mortality rates (27.53 for Oceania, 16.60 for Central Asia, 11.18 for South Asia) and DALY rates (2,421.89 for Oceania, 1,461.52 for Central Asia, 987.25 for South Asia) from LRIs attribute to SHS, with the lowest rates in under-5s of mortality and DALYs in Western Europe (mortality rate: 0.19, DALYs rate: 17.55), Australasia (mortality rate: 0.24, DALYs rate: 21.55), and high-income North America (mortality rate: 0.35, DALYs rate: 31.45) (Table 1). From 2010 to 2019, all regions showed a decrease in the under-5 mortality rates from LRIs attributed to SHS, with the largest decreases in East Asia (− 9.72%), Central Sub-Saharan Africa (− 9.07%), and Eastern Europe (− 7.44%) (Table 1). The DALY rates in under-5s also decreased in all regions from 2010 to 2019, with the largest decreases also in East Asia (− 9.70%), Central Sub-Saharan Africa (− 9.05%), and Eastern Europe (− 7.40%).

The percentage of deaths and DALYs caused by LRIs attributable to SHS slightly decreased or even remained stagnant from 2010 to 2019 globally among children under 5 years old. This pattern was observed in most regions, with the exception of Andean Latin America, the Caribbean, high-income North America, Oceania, South Asia, Southeast Asia, Tropical Latin America, and Western Sub-Saharan Africa, for which percentages were slightly increased from 2010 to 2019. Noticeably, SHS was attributed to a large extent of the under-5 LRIs burden in Eastern Europe (20.78%), East Asia (19.59%), and Central Europe (18.16%) in 2019.

### National level

**Fig. 1:**
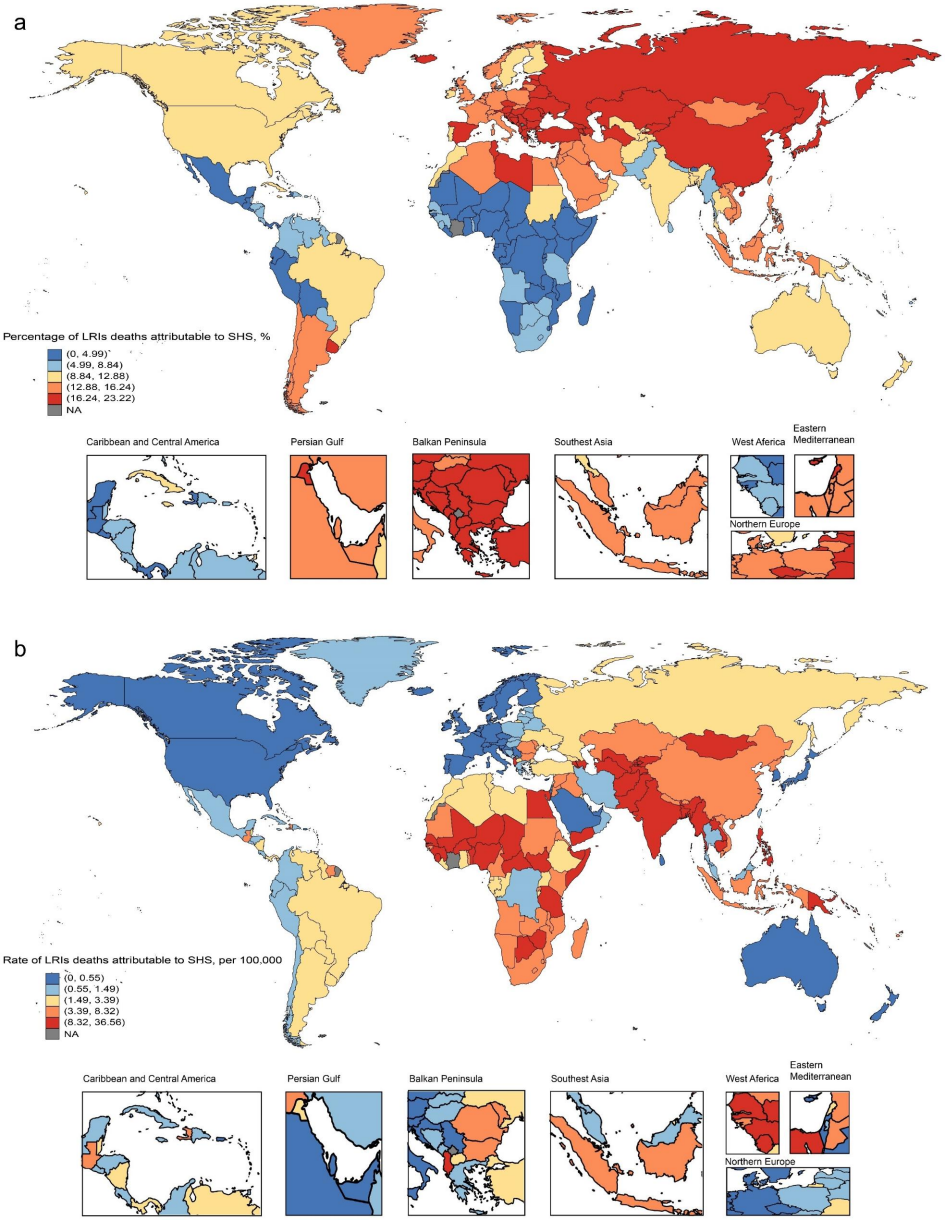
Global burden of LRIs deaths attributable to SHS among children under 5 years of age in 2019. **(a)** Percentage of LRIs deaths attributable to SHS; **(b)** Rate of LRIs deaths attributable to SHS. LRIs: lower respiratory infections; SHS: secondhand smoke

In 2019, the national under-5 mortality rates for LRIs varied from 0.05 to 36.56 per 100,000. The highest rates were seen in Azerbaijan (36.56), Turkmenistan (32.90), and Papua New Guinea (32.24), whereas the lowest rates were found in Finland (0.05), Norway (0.07), and Sweden (0.09) (Fig. 1B, Table S1). In 2019, the national DALY rate in under-5s of LRIs ranged from 7.66 to 5838.06 patients per 100,000. The highest rates were also seen in Azerbaijan (3224.34), Turkmenistan (2890.91), and Papua New Guinea (2836.02), whereas the lowest rates were in Finland (5.39), Norway (7.37), and Sweden (9.25) (Figure S1B, Table S1). The percentage change in the under-5 mortality rate, from 2010 to 2019, differed noticeably between countries, with Iran (Islamic Republic of) (16.09%), Saudi Arabia (11.04%), and Cook Islands (10.57%) having the largest decreases. In contrast, the Northern Mariana Islands (2.79%), Dominica (2.71%), Burkina Faso (0.32%), Ukraine (0.14%), and Niger (0.06%) had increasing trends (Table S1). Over the same period, Iran (Islamic Republic of) (16.03%), Saudi Arabia (10.66%), and Cook Islands (10.50%) had the largest decreases in the DLAYs rate, whereas increases were also found in the Northern Mariana Islands (2.81%), Dominica (2.72%), Burkina Faso (0.32%), Ukraine (0.16%), and Niger (0.07%). In 2019, Bosnia and Herzegovina (23.22%), Armenia (23.12%), and Montenegro (23.07%) showed the highest percentage of LRIs burden due to SHS, whereas Sao Tome and Principe (1.33%), Ethiopia (1.35%), and the Democratic Republic of the Congo (1.37%) showed distinctly small extent of this burden (Fig. 1A, Figure S1A, Table S1).

### Age-sex pattern and temporal trend

**Fig. 2:**
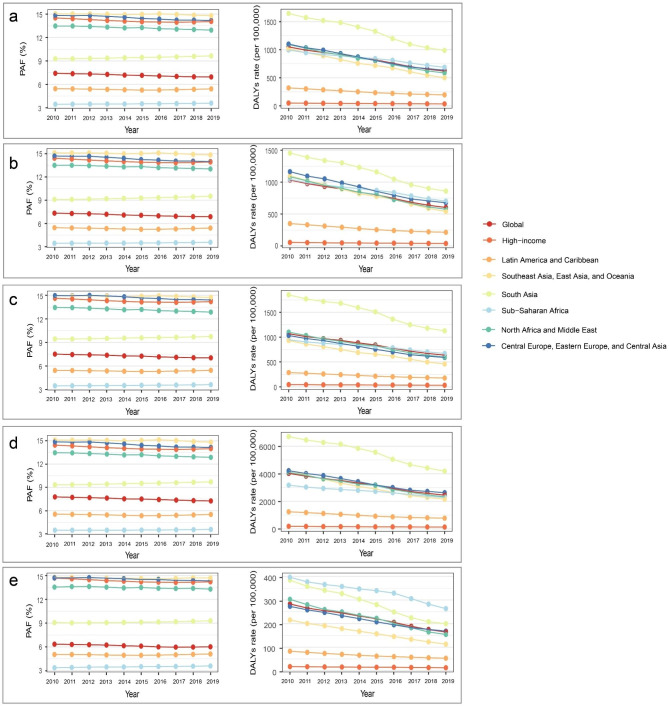
Temporal trend in LRIs DALYs attributable to SHS among children under 5 years of age, 2010–2019. **(a)** Data are for all ages and both sexes by GBD super-region and globally; **(b)** Data are for male; **(c)** Data are for female; **(d)** Data are for < 1 year old; **(e)** Data are for 1–5 years old. LRIs: lower respiratory infections; SHS: secondhand smoke; DALYs: disability-adjusted life-years; PAF: population attributable fraction

Globally, we observed slight decreases in the PAF of DALYs from 2010 to 2019 (Fig. 2A), and the PAF was higher in children < 1 year than in children 1–4 years (Fig. 2B C). This pattern was observed for most regions, with the exception of South Asia and Sub − Saharan Africa, for which PAF were slightly increased after 2010. With regard to the time trend in the DALYs rate of LRIs attributable to SHS, we found a decrease in most super-regions from 2010 to 2019, whereas the rate of DALYs were stagnant and remained at the lowest level over time in high − income regions. The rate of DALYs is much greater in children younger than 1 year than in children aged 1–4 years. The highest rate of DALYs among children under 1 year of age was observed in South Asia, while the highest rate of DALYs among children aged 1–4 years was shown in Sub-Saharan Africa. With the exception of South Asia, which shows a higher DALYs rate in females, other regions show a similar pattern of the LRIs burden attributable to SHS between females and males (Fig. 2D and E). Regardless of age-sex pattern or time period, the PAFs and rates for DALYs in Latin America and Caribbean are lower than global levels. However, in high-income regions, PAF levels are remarkably above global levels despite low rates of DALYs in under-5s (Fig. 2A).

### Association with the SDI

**Fig. 3:**
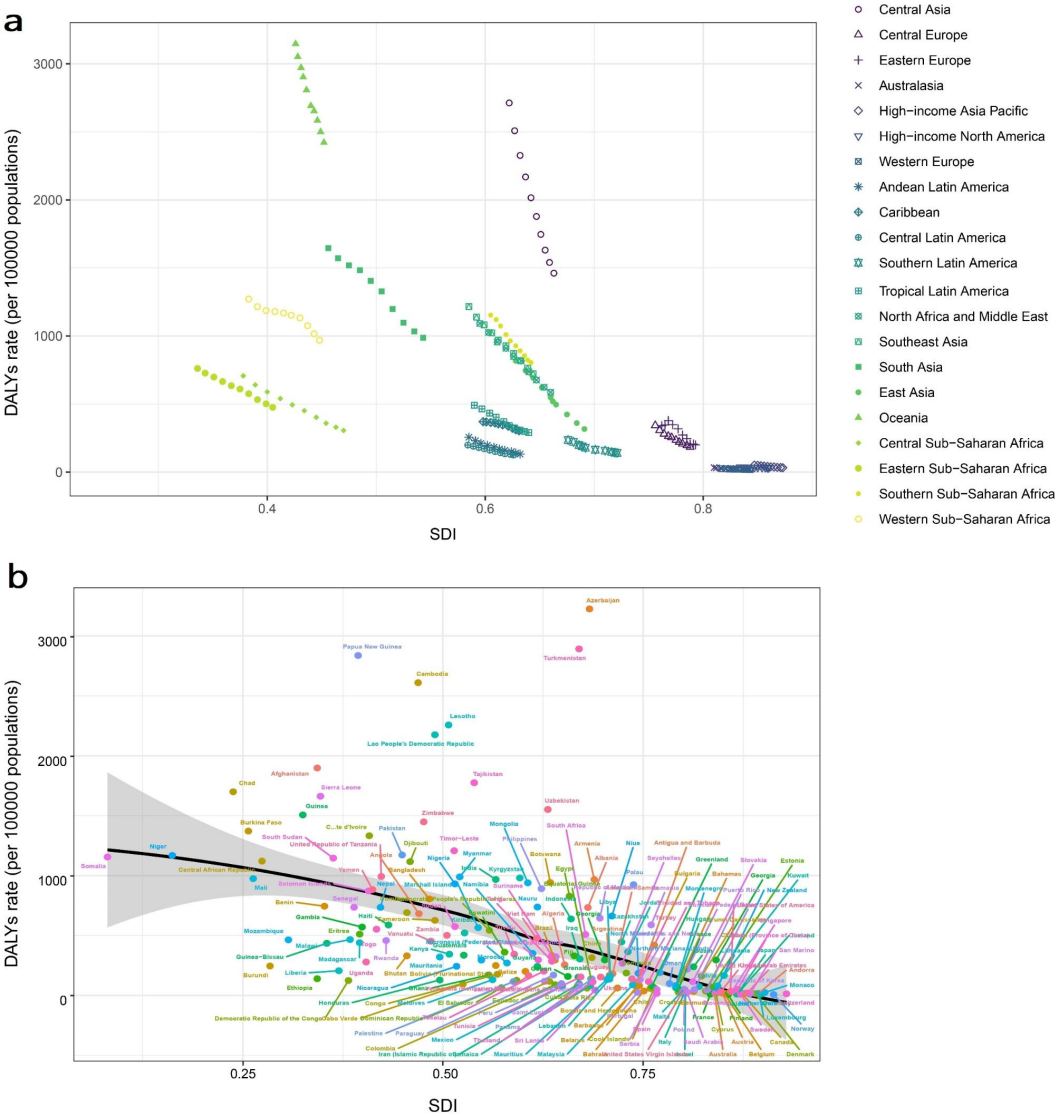
The correlation between SDI and DALYs rate of LRIs attributable to SHS among children under 5 years of age, 2010–2019. **(a)** In 21 GBD regions and five SDI regions; **(b)** In 204 countries and territories. SDI: sociodemographic index; DALYs: disability-adjusted life-years; LRIs: lower respiratory infections; SHS: secondhand smoke

At the regional level, we found a negative association between the SDI and the DALYs rate of LRIs in uner-5s from 2010 to 2019 (Fig. 3), as well as the SDI and mortality rate (Figure S2). Except for some regions with high SDI, such as Australasia, Western Europe and high-income Asia Pacific, where the under-5 mortality and DALYs rate remained stable, low- and middle-SDI countries showed large decreases in the rate of mortality and DALYs from 2010 to 2019 with the development of SDI. Oceania and Central Asia had higher level than the other regions based on their SDIs, from 2010 to 2019 (Fig. 3A, Figure S2A). At the country level, in 2019, the burden of LRIs decreased with increasing socioeconomic development up to SDI. Countries and territories such as Azerbaijan, Turkmenistan, Papua New Guinea, and Cambodia had much higher than expected burdens, whereas Burundi, Liberia, Ethiopia, and the Democratic Republic of the Congo had much lower than expected burdens (Fig. 3B, Figure S2B).

## Discussion

### Principal findings

In this study, based on data from the GBD 2019 study, we provided up-to-date information on the LRIs burden (deaths and DALYs) attributable to SHS in children under 5 years of age from 2010 to 2019 across 204 countries and territories. Approximately one-seventh of the global burden of LRIs was attributable to SHS in 2019, with 6.94% for deaths, and 6.95% for DALYs. Although the under-5 mortality and DALYs rates of LRIs due to SHS have decreased over the past ten years, the PAF was stagnant and there is even a year-on-year upward trend in South Asia. Moreover, the burden was heavier in children under one year of age and was significantly associated with SDI.

### Comparison with other studies

Despite the substantial progress made in children younger than 5 years, there were still 672,000 LRI deaths in this age group in 2019, 93.5% (95% UIs 90.4–95.7) of those deaths were attributable to preventable risk factors, and SHS was a top tenth evaluated risk factor for PAFs of LRI deaths (approximately 7%) among males and females in 2019 globally [6]. Although the burden rate of LRIs attributable to SHS estimates were not reported in this previous study, the overall results for the PAFs of LRI death were consistent with our findings, which shows that PAF was highest in Southeast Asia, East Asia, and Oceania. In addition, based on our study, the DALYs rate in South Asia is much higher than the global level, which shows that the burden of LRIs attributable to SHS among children under 5 years was heavier in Asia. Although smoking bans have been increasingly applied all over the world after the recommendation of the WHO in 2007 [16] to comply with Article 8 of the Framework Convention on Tobacco Control (FCTC), some Asian countries do not have smoke-free environments at home, work, and in public places [21]. In 2015, China, India, and Indonesia had the largest number of smokers [22] and many non-smokers in Asia tend to be highly exposed to SHS. Because their bodies are still growing, infants and young children are especially vulnerable to respiratory illness risks from SHS such as LRIs [23]. No amount of SHS is safe [23] and children cannot choose their own living environment. Though with the development of economics, the burden due to LRI attributable to SHS exposure among children under 5 years also decreased in Asia from 2010 to 2019 at the region level; however, some middle-income countries, such as Azerbaijan, Turkmenistan, and Cambodia, the burden of LRIs attributed to SHS in children under five years of age is much higher than the average in other middle-income countries, morbidity caused by SHS exposure is not negligible and calls for further public health awareness.

The lowest burden rate of LRIs attributable to SHS among children under 5 years of age was observed in high-income regions in our study, especially the Western Europe, such as Finland, Norway, and Sweden. Since smoke-free policies (SFPs) has been broadly applied in workplaces, public venues and transportation [15]. The social unacceptability of SHS and consequently the adoption of voluntary smoking bans in homes in European Union countries increased [24]. Available evidence has shown that as early as 2010, the Finnish government announced that it was hoping to impose a complete ban on smoking by 2040. However, now, the date for the ban has been brought forward by 10 years: in 2030, the government plans to start to phase out all tobacco products. Only 15.4.% of the Finnish population are classified as daily smokers, according to an OECD study from 2015, which puts them on an equal footing with their Swedish and Norwegian neighbor [25]. For other high-income countries, a study conducted in Spain showed that the PAF_SHS_ estimated for LRI ranged from 11.1 to 27.6% in 2015. The markedly highest PAF_SHS_ were found for bronchiolitis in infants 0 to 1 years old, which doubled those obtained for bronchitis and LRI in general [12]. Another study conducted in the United Kingdom estimated that the disease events caused by SHS exposure at home in children in 2008 and reported that PAF_SHS_ of LRI was 11% [13]. These estimates seem to differ from our results, which is considerably higher than those in these studies. However, comparisons between the studies should be interpreted with caution, due to methodological differences such as the year when the study was conducted, the age groups analyzed, sources of data, differences in SHS exposure measurement, and studied health outcomes, for example, our study focused on the impact of SHS on the burden of LRI disease, while other studies looked at the impact of SHS on the incidence of LRIs.

In contrast, low- and middle-income countries such as those in Africa continue to lag behind high-income countries [26–28]. Only a handful of African countries have comprehensive, or 100%, SFPs covering all public and privates places, including restaurants, bars, and night clubs [26, 28] and as a region, Africa is projected to have the most rapid growth in tobacco smoking by 2025 [29]. Though Sub − Saharan Africa has the lowest PAF_SHS_ of LRIs DALYs, highest DALYs rate was observed among children in 5 years compared with other GBD super regions in the world. The reasons for this need to be analyzed from the proportion of factors contributing to the burden of disease in LRIs in Africa. According to previous studies [6], the impact of household air pollution and poor handwashing in Sub − Saharan Africa for LRIs burden is higher than that on SHS burden. However, the adoption and implementation of evidence-based policy initiatives to reduce the LRIs burden attributable to SHS are still paramount. It was reported that children under 5 years of age living in deprived communities were more likely to be exposed to SHS in the home. And according to the monotonic relation between DALYs due to LRIs attributable to SHS and SDI at the national level, some Sub − Saharan Africa countries, like Somalia and Niger, with low SDI scores, were estimated to have the higher burden of LRIs attributable to SHS in 2019. A previous study has showed that countries with a lower SDI generally have poor access to healthcare services, including limited availability and affordability of diagnostic and therapeutic measures [30]. Therefore, the burden of LRIs attributable to SHS among children under 5 years remained a serious problem in Sub − Saharan Africa.

Although there had been a general decline in the global burden of LRIs attributable to SHS in under-5s in most regions from 2010 to 2019, the PAF remained relatively stable. This might be due to the fact that the overall disease burden caused by LRI has decreased globally [5], but SHS exposure remains constant or changes only slightly. However, it is worth mentioning that the Latin America and the Caribbean not only has a relatively low burden of SHS-LRI, but also PAF has been at a low level from 2010 to 2019. Substantial progress in Latin America and the Caribbean with regards to SHS control can be attributed to the implementation of strong tobacco control policies. The adoption of FCTC Article 8, which focuses on protecting individuals from exposure to SHS, has been effectively carried out in Latin America through the Smokefree Americas Initiative. Launched by the Pan American Health Organization in 2001, this initiative has been made possible by a combination of five key factors: the emergence of dedicated advocacy groups in the region, the establishment of a coordinated network of tobacco control advocates and researchers, collaboration between the government and civil society, technical support provided by international organizations, and the availability of funding from developed countries to support tobacco control efforts in Latin America [31]. These measures may have significant implications for implementing SHS exposure reduction plans in other regions with high PAF_SHS_ of LRIs burden. According to our results, a more continuous and consistent surveillance of secondhand smoking in Asia and European regions is needed, as well as strengthening efforts to better control tobacco use. This could involve the formulation and enforcement of stringent smoke-free legislation in public spaces, educational institutions, and indoor areas where children frequent. Implementing comprehensive public awareness campaigns would be crucial in elucidating the detrimental health consequences of SHS and fostering a societal aversion to smoking around children. Moreover, governments could consider providing accessible and affordable smoking cessation programs for parents and caregivers, addressing the root cause of SHS exposure. Collaborative efforts between healthcare providers and schools can lead to educational initiatives that highlight the risks of SHS, encouraging parents to adopt smoke-free homes. Health systems could incorporate routine screenings for SHS exposure during pediatric visits, allowing for early detection and intervention.

### Strengths and limitations of this study

A strength of the study is that we have provided up-to-date and comprehensive estimates of levels and trends associated with LRIs attributable to SHS among children under 5 years at the global, regional, and national levels, between 2010 and 2019. The study had several limitations. First, data on SHS were self-reported, which might be prone to underestimation depending on respondents’ perception of the social acceptance of smoking and recall bias, so there may be errors in the burden of disease attributed to SHS. Second, the data used in this study were derived from the GBD database, which only collected public data and did not include the unpublished data. Third, the GBD data are only updated to 2019, and thus, we cannot obtain data from the last three years. The impact of COVID-19 on LRIs burden in children under 5 years of age attributable to SHS cannot be estimated. Forth, as a secondary analysis of the GBD data, we have no additional detailed covariable data to control the bias and the accuracy and robustness of the GBD estimate largely depend on the quality and quantity of data used in the modeling. Finally, the lack of original data in some regions and the fact that some of the numbers are just an estimation also is one of the limitations of the GBD study.

## Conclusions

Although the burden of LRIs attributable to SHS in children under 5 years of age has been substantially improved globally in recent decades, the progress has varied across countries. The incidence and DALY rate were closely associated with the SDI of a country. The burden of LRIs attributable to SHS in children under 5 years of age in certain low and middle socioeconomic countries remain worrisome. This knowledge could guide policy makers in planning control SHS measures and supply services to meet the rising healthcare demands that LRIs and its comorbidities will create.

### Electronic supplementary material

Below is the link to the electronic supplementary material.

Supplementary Material 1

## Data Availability

The data in this article are available in the GBD 2019 database at http://ghdx.healthdata.org/gbd-results-tool (accessed on 1 October 2022).

## Abbreviations

GBD: Global burden of disease
LRIs: Lower respiratory infections
SHS: Secondhand smoke
YLL: Years of healthy life lost due to premature death
YLD: Years lived with a disability
DALYs: Disability adjusted life years
PAF: Population-attributable fraction
EAPC: Estimated the annual percentage change
SDI: Sociodemographic index
ICD: International classification of diseases
TMREL: Theoretical minimum risk exposure level
UIs: Uncertainty intervals
WHO: World health organization
FCTC: Framework convention on tobacco control
SFPs: Smoke-free policies
