# Supplementary material for: Global burden of lower respiratory infections attributable to secondhand smoke among children under 5 years of age, 2010–2019: a systematic analysis of the global burden of disease study 2019
Source: BMC Public Health. 2023 Oct 4;23:1920. doi: 10.1186/s12889-023-16848-5 (PMC10552277; doi:10.1186/s12889-023-16848-5)
Supplement: Supplementary file 1 — Supplementary Material 1 [file 12889_2023_16848_MOESM1_ESM.docx]

Table S1. LRIs deaths and DALYs attribute to SHS among children under 5 years of age in 204 countries 2010 to 2019.

|  | Deaths | | | | |  | DALYs | | | | |
| --- | --- | --- | --- | --- | --- | --- | --- | --- | --- | --- | --- |
|  | 2010 | | 2019 | | 2010-2019 |  | 2010 | | 2019 | | 2010-2019 |
|  | Percentage, % | Rate, 10^5^ | Percentage, % | Rate, 10^5^ | EAPC, % |  | Percentage, % | Rate, 10^5^ | Percentage, % | Rate, 10^5^ | EAPC, % |
| Afghanistan | 10.03 (5.98 to 14.38) | 36.67 (19.8 to 58.77) | 10.38 (6.21 to 14.98) | 21.52 (11.43 to 34.72) | -5.58% (-5.92 to -5.23) |  | 10.03 (5.99 to 14.39) | 3,231.75 (1,743.85 to 5,172.89) | 10.38 (6.21 to 14.98) | 1,897.44 (1,008.9 to 3,055.79) | -5.57% (-5.92 to -5.23) |
| Albania | 20.82 (13.82 to 27.82) | 10.59 (6.8 to 14.7) | 20.88 (14.19 to 28.02) | 8.38 (5.38 to 12.06) | -2.51% (-3.37 to -1.65) |  | 20.82 (13.82 to 27.82) | 931.01 (597.45 to 1,291.44) | 20.88 (14.18 to 28.04) | 733.3 (472.02 to 1,051.96) | -2.57% (-3.4 to -1.73) |
| Algeria | 16.02 (10.39 to 21.84) | 5.57 (3.21 to 8.38) | 16.13 (10.28 to 22.06) | 2.88 (1.68 to 4.38) | -6.93% (-7.71 to -6.15) |  | 16.02 (10.39 to 21.84) | 492.46 (284.26 to 740.22) | 16.13 (10.28 to 22.06) | 255.04 (148.85 to 388.1) | -6.91% (-7.68 to -6.13) |
| American Samoa | 10.6 (5.71 to 15.87) | 2.61 (1.33 to 4.26) | 10.23 (5.57 to 15.39) | 2.05 (0.98 to 3.54) | -2.18% (-2.65 to -1.71) |  | 10.6 (5.71 to 15.87) | 230.09 (117.74 to 376.3) | 10.23 (5.57 to 15.39) | 181.03 (86.9 to 312.63) | -2.17% (-2.64 to -1.7) |
| Andorra | 16.72 (10.85 to 23.09) | 0.35 (0.2 to 0.52) | 15.7 (9.97 to 21.89) | 0.18 (0.1 to 0.29) | -7.39% (-9.26 to -5.48) |  | 16.72 (10.85 to 23.07) | 31.43 (18.42 to 46.9) | 15.69 (9.93 to 21.87) | 17.05 (9.19 to 26.59) | -7.08% (-8.88 to -5.24) |
| Angola | 7.09 (3.92 to 10.61) | 19.66 (10.17 to 30.31) | 7.16 (3.98 to 10.64) | 7.75 (3.9 to 12.4) | -10.01% (-10.27 to -9.76) |  | 7.09 (3.92 to 10.6) | 1,725.24 (892.27 to 2,656.98) | 7.16 (3.98 to 10.63) | 681.89 (342.7 to 1,091.22) | -9.99% (-10.24 to -9.74) |
| Antigua and Barbuda | 6.6 (3.88 to 9.5) | 1.58 (0.87 to 2.46) | 6.43 (3.77 to 9.41) | 1.26 (0.66 to 2.15) | -2.44% (-2.75 to -2.14) |  | 6.6 (3.89 to 9.5) | 139.31 (76.81 to 216.51) | 6.43 (3.77 to 9.41) | 111.35 (57.81 to 188.94) | -2.44% (-2.73 to -2.14) |
| Argentina | 15.13 (9.15 to 21.33) | 3.17 (1.89 to 4.49) | 14.57 (8.7 to 20.86) | 1.89 (1.03 to 2.98) | -5.82% (-6.42 to -5.22) |  | 15.13 (9.15 to 21.33) | 280.38 (167.85 to 397.46) | 14.57 (8.7 to 20.86) | 167.72 (91.48 to 263.34) | -5.81% (-6.41 to -5.2) |
| Armenia | 22.76 (15.33 to 30.22) | 18.2 (11.97 to 25.39) | 23.12 (15.55 to 30.64) | 10.99 (6.84 to 15.96) | -5.38% (-5.82 to -4.93) |  | 22.76 (15.33 to 30.22) | 1,603.27 (1,055.09 to 2,235.53) | 23.12 (15.54 to 30.63) | 966.7 (602.6 to 1,406.07) | -5.39% (-5.83 to -4.94) |
| Australia | 13.43 (8.1 to 18.99) | 0.34 (0.2 to 0.5) | 12.35 (7.37 to 17.9) | 0.22 (0.13 to 0.35) | -4.1% (-4.61 to -3.59) |  | 13.43 (8.11 to 19.04) | 30.75 (18.06 to 45.8) | 12.35 (7.37 to 17.9) | 20.35 (11.56 to 31.68) | -4.08% (-4.57 to -3.59) |
| Austria | 20.09 (13.37 to 26.93) | 0.31 (0.19 to 0.43) | 19.91 (13.49 to 26.8) | 0.19 (0.12 to 0.28) | -3.89% (-5.56 to -2.19) |  | 20.09 (13.36 to 26.93) | 29.26 (18.42 to 40.77) | 19.91 (13.49 to 26.68) | 18.72 (11.5 to 26.95) | -3.65% (-5.21 to -2.06) |
| Azerbaijan | 21.19 (14.27 to 28.26) | 66.2 (42.39 to 93.08) | 21.12 (14.42 to 28.07) | 36.56 (21.8 to 55.81) | -6.48% (-6.64 to -6.32) |  | 21.19 (14.27 to 28.26) | 5,838.06 (3,733.96 to 8,206.5) | 21.12 (14.42 to 28.06) | 3,224.34 (1,925.52 to 4,917.03) | -6.48% (-6.64 to -6.32) |
| Bahamas | 8.1 (4.84 to 11.52) | 1.91 (1.06 to 2.87) | 7.68 (4.51 to 11.07) | 1.49 (0.82 to 2.41) | -2.52% (-2.71 to -2.34) |  | 8.1 (4.84 to 11.52) | 168.61 (93.81 to 252.66) | 7.68 (4.51 to 11.07) | 131.48 (72.72 to 213.45) | -2.53% (-2.71 to -2.34) |
| Bahrain | 12.91 (8.21 to 17.97) | 0.7 (0.43 to 1.03) | 12.89 (7.99 to 17.93) | 0.41 (0.23 to 0.64) | -5.74% (-6.69 to -4.78) |  | 12.91 (8.21 to 17.96) | 63.08 (38.63 to 91.84) | 12.89 (8 to 17.92) | 37.19 (21.31 to 57.75) | -5.59% (-6.5 to -4.68) |
| Bangladesh | 9.69 (4.72 to 15.36) | 17.61 (8.09 to 29.69) | 10.18 (5.12 to 15.83) | 9.11 (4.12 to 15.36) | -7.13% (-7.32 to -6.94) |  | 9.69 (4.72 to 15.36) | 1,554.05 (713.38 to 2,619.9) | 10.18 (5.12 to 15.82) | 804.44 (363.66 to 1,355.37) | -7.12% (-7.31 to -6.93) |
| Barbados | 5.94 (3.4 to 8.65) | 1.06 (0.57 to 1.63) | 5.51 (3.05 to 8.22) | 0.83 (0.4 to 1.4) | -2.66% (-3.24 to -2.07) |  | 5.94 (3.4 to 8.65) | 93.88 (50.42 to 145.04) | 5.51 (3.05 to 8.22) | 73.88 (35.76 to 123.95) | -2.64% (-3.23 to -2.06) |
| Belarus | 19.56 (12.51 to 26.46) | 1.68 (1 to 2.46) | 20.66 (13.61 to 27.63) | 0.91 (0.52 to 1.4) | -6.36% (-6.76 to -5.96) |  | 19.56 (12.51 to 26.46) | 149.47 (88.68 to 219.29) | 20.66 (13.61 to 27.63) | 81.79 (46.68 to 124.64) | -6.28% (-6.69 to -5.87) |
| Belgium | 15.41 (10.13 to 20.85) | 0.29 (0.19 to 0.42) | 14.86 (9.38 to 20.56) | 0.2 (0.12 to 0.31) | -4.12% (-4.48 to -3.76) |  | 15.4 (10.11 to 20.86) | 27.7 (17.88 to 39.05) | 14.86 (9.38 to 20.56) | 19.01 (11.47 to 28.6) | -4.02% (-4.35 to -3.7) |
| Belize | 6.86 (3.76 to 10.31) | 2.98 (1.56 to 4.71) | 7.08 (3.89 to 10.52) | 2.27 (1.15 to 3.7) | -2.99% (-3.21 to -2.78) |  | 6.86 (3.76 to 10.31) | 263.18 (137.88 to 416.56) | 7.08 (3.89 to 10.53) | 200.42 (101.8 to 326.56) | -2.99% (-3.21 to -2.77) |
| Benin | 3.55 (1.75 to 5.63) | 12.24 (5.6 to 21.11) | 3.64 (1.84 to 5.81) | 8.49 (3.64 to 15.35) | -3.58% (-4.44 to -2.72) |  | 3.55 (1.74 to 5.63) | 1,073.16 (490.5 to 1,847.7) | 3.64 (1.84 to 5.81) | 744.32 (319.92 to 1,342.77) | -3.58% (-4.43 to -2.72) |
| Bermuda | 7.47 (4.24 to 11.05) | 0.44 (0.24 to 0.7) | 7.86 (4.52 to 11.4) | 0.35 (0.18 to 0.57) | -2.4% (-2.7 to -2.1) |  | 7.47 (4.24 to 11.04) | 39.09 (21.17 to 62.5) | 7.86 (4.53 to 11.41) | 31.08 (16.47 to 50.35) | -2.35% (-2.66 to -2.04) |
| Bhutan | 4.62 (2.51 to 6.96) | 6.87 (3.11 to 11.78) | 4.96 (2.75 to 7.55) | 3.75 (1.62 to 6.8) | -6.3% (-6.8 to -5.79) |  | 4.62 (2.52 to 6.96) | 605.35 (273.78 to 1,035.98) | 4.96 (2.75 to 7.56) | 330.71 (142.8 to 597.97) | -6.29% (-6.79 to -5.79) |
| Bolivia (Plurinational State of) | 2.59 (0.89 to 4.86) | 5.39 (1.81 to 10.66) | 2.71 (0.93 to 5.06) | 2.83 (0.91 to 5.34) | -6.96% (-7.11 to -6.81) |  | 2.59 (0.89 to 4.86) | 473.95 (159.33 to 936.19) | 2.71 (0.93 to 5.06) | 249.23 (80.41 to 470.1) | -6.96% (-7.11 to -6.81) |
| Bosnia and Herzegovina | 24.16 (16.33 to 31.86) | 0.82 (0.52 to 1.17) | 23.22 (15.6 to 30.76) | 0.67 (0.41 to 1.02) | -0.6% (-2.6 to 1.44) |  | 24.16 (16.33 to 31.86) | 74.37 (48.36 to 105.93) | 23.22 (15.6 to 30.76) | 61.89 (37.74 to 93.16) | -0.52% (-2.45 to 1.44) |
| Botswana | 9.44 (4.84 to 14.49) | 14.04 (6.41 to 23.39) | 8.84 (4.44 to 14.03) | 10.75 (4.69 to 19.33) | -3.02% (-3.16 to -2.89) |  | 9.44 (4.84 to 14.5) | 1,231.04 (562.54 to 2,052.22) | 8.84 (4.44 to 14.04) | 941.79 (412.42 to 1,692.16) | -3.03% (-3.17 to -2.89) |
| Brazil | 9.85 (5.66 to 14.5) | 5.68 (3.19 to 8.66) | 10.61 (6.51 to 15.18) | 3.34 (1.88 to 4.96) | -6.04% (-6.59 to -5.49) |  | 9.85 (5.66 to 14.51) | 500.47 (281.37 to 762.84) | 10.61 (6.5 to 15.18) | 294.96 (165.69 to 437.61) | -6.03% (-6.58 to -5.48) |
| Brunei Darussalam | 15.2 (9.47 to 21.18) | 1.92 (1.17 to 2.7) | 14.46 (9.05 to 20.07) | 1.81 (1.05 to 2.8) | -1.25% (-2.52 to 0.03) |  | 15.2 (9.46 to 21.17) | 170.06 (103.79 to 239.3) | 14.46 (9.05 to 20.06) | 160.37 (93.15 to 247.64) | -1.25% (-2.51 to 0.02) |
| Bulgaria | 24.68 (16.66 to 32.48) | 10.42 (6.98 to 13.91) | 22.63 (14.81 to 30.24) | 4.73 (2.78 to 6.86) | -7.89% (-9.5 to -6.26) |  | 24.68 (16.66 to 32.48) | 920.72 (616.11 to 1,228.91) | 22.63 (14.81 to 30.24) | 419.33 (246.19 to 606.16) | -7.86% (-9.46 to -6.24) |
| Burkina Faso | 3.57 (1.4 to 6.5) | 15.56 (5.55 to 30.9) | 3.83 (1.61 to 6.74) | 15.64 (5.81 to 30.06) | 0.32% (0.01 to 0.64) |  | 3.57 (1.4 to 6.5) | 1,363.9 (486.89 to 2,711.56) | 3.83 (1.61 to 6.74) | 1,371.16 (510.25 to 2,633.07) | 0.32% (0.01 to 0.64) |
| Burundi | 2.18 (0.79 to 4.05) | 4.75 (1.36 to 10.39) | 2.29 (0.83 to 4.25) | 2.79 (0.83 to 6.43) | -6.22% (-7.31 to -5.12) |  | 2.18 (0.79 to 4.05) | 417.8 (119.4 to 912.89) | 2.29 (0.83 to 4.25) | 245.35 (73.09 to 566.02) | -6.21% (-7.29 to -5.11) |
| Cabo Verde | 3.83 (1.84 to 6.23) | 2.02 (0.86 to 3.62) | 3.9 (1.85 to 6.27) | 1.08 (0.36 to 2.11) | -5.99% (-7.08 to -4.9) |  | 3.83 (1.84 to 6.23) | 178.64 (75.92 to 319.08) | 3.9 (1.85 to 6.27) | 95.22 (32.26 to 186.34) | -6% (-7.07 to -4.91) |
| Cambodia | 15.14 (9.31 to 20.79) | 52.68 (31.53 to 76.97) | 15.64 (9.81 to 21.75) | 29.58 (17.1 to 45.12) | -6.01% (-6.3 to -5.73) |  | 15.14 (9.31 to 20.79) | 4,645.3 (2,780.83 to 6,789.68) | 15.64 (9.81 to 21.74) | 2,609.02 (1,507.85 to 3,980.44) | -6.01% (-6.29 to -5.73) |
| Cameroon | 4.14 (2.06 to 6.55) | 12.03 (5.46 to 21.77) | 3.84 (1.84 to 6.2) | 7.13 (3.03 to 12.81) | -5.82% (-6.13 to -5.51) |  | 4.14 (2.06 to 6.55) | 1,055.31 (479.04 to 1,910.47) | 3.84 (1.84 to 6.2) | 626.29 (266.96 to 1,127.21) | -5.81% (-6.11 to -5.5) |
| Canada | 12.76 (8.28 to 18.04) | 0.24 (0.14 to 0.34) | 12.72 (8.32 to 17.8) | 0.2 (0.13 to 0.3) | -1.35% (-2.22 to -0.48) |  | 12.76 (8.28 to 18.02) | 21.45 (13.28 to 31.17) | 12.72 (8.31 to 17.81) | 18.49 (11.51 to 27.13) | -1.33% (-2.15 to -0.49) |
| Central African Republic | 3.05 (1.36 to 5.2) | 17.14 (6.71 to 32.77) | 3.28 (1.49 to 5.45) | 12.75 (5.06 to 24.15) | -2.96% (-3.34 to -2.58) |  | 3.05 (1.36 to 5.2) | 1,504.95 (588.6 to 2,879.87) | 3.28 (1.49 to 5.45) | 1,120.3 (443.78 to 2,121.75) | -2.95% (-3.33 to -2.58) |
| Chad | 4.73 (2.1 to 7.96) | 26.53 (10.7 to 47.86) | 4.69 (2.16 to 7.76) | 19.37 (7.88 to 34.45) | -3.36% (-3.52 to -3.2) |  | 4.73 (2.1 to 7.96) | 2,324.13 (937.95 to 4,195.79) | 4.69 (2.16 to 7.76) | 1,699.05 (690.72 to 3,022.73) | -3.35% (-3.51 to -3.19) |
| Chile | 12.89 (6.85 to 19.47) | 1.08 (0.55 to 1.7) | 12.92 (6.92 to 19.32) | 0.64 (0.3 to 1.08) | -5.52% (-6.2 to -4.84) |  | 12.89 (6.85 to 19.46) | 96.67 (50.07 to 153.1) | 12.92 (6.92 to 19.32) | 57.97 (27.14 to 96.53) | -5.48% (-6.14 to -4.81) |
| China | 19.82 (13.17 to 26.53) | 9.25 (5.93 to 12.38) | 19.66 (13.06 to 26.42) | 3.56 (2.26 to 4.91) | -9.74% (-10.61 to -8.87) |  | 19.82 (13.17 to 26.53) | 817.68 (523.74 to 1,094.28) | 19.66 (13.06 to 26.43) | 315 (200.65 to 435.02) | -9.73% (-10.59 to -8.85) |
| Colombia | 4.75 (2.06 to 8.04) | 1.87 (0.75 to 3.3) | 5.12 (2.39 to 8.42) | 1.32 (0.54 to 2.47) | -4.05% (-4.59 to -3.52) |  | 4.75 (2.06 to 8.04) | 165.31 (66.24 to 290.83) | 5.12 (2.39 to 8.42) | 116.94 (48.12 to 217.97) | -4.03% (-4.56 to -3.5) |
| Comoros | 4.96 (2.33 to 8.1) | 14.13 (6.28 to 26.01) | 5.01 (2.36 to 8.1) | 7.84 (3.35 to 14.21) | -6.28% (-6.8 to -5.75) |  | 4.96 (2.33 to 8.1) | 1,244.61 (553.41 to 2,298.74) | 5.01 (2.36 to 8.1) | 690.9 (294.17 to 1,252.38) | -6.27% (-6.79 to -5.75) |
| Congo | 4.07 (1.91 to 6.59) | 4.18 (1.73 to 7.65) | 4.17 (1.98 to 6.71) | 2 (0.84 to 3.64) | -8.07% (-8.4 to -7.74) |  | 4.07 (1.91 to 6.6) | 367.84 (152.44 to 673.09) | 4.17 (1.98 to 6.71) | 176.46 (74.4 to 321.37) | -8.05% (-8.38 to -7.72) |
| Cook Islands | 10.72 (5.49 to 16.54) | 2.17 (0.95 to 3.87) | 11.2 (5.97 to 16.86) | 0.73 (0.3 to 1.33) | -10.57% (-12.63 to -8.47) |  | 10.72 (5.5 to 16.55) | 190.76 (83.7 to 338.86) | 11.2 (5.97 to 16.85) | 65.1 (27.27 to 117.44) | -10.5% (-12.54 to -8.4) |
| Costa Rica | 7.84 (4.31 to 11.7) | 0.87 (0.43 to 1.42) | 8.16 (4.51 to 11.95) | 0.68 (0.32 to 1.19) | -2.15% (-3.11 to -1.18) |  | 7.84 (4.31 to 11.7) | 77.46 (39 to 126.53) | 8.16 (4.5 to 11.95) | 60.56 (28.64 to 106.21) | -2.14% (-3.08 to -1.19) |
| Croatia | 21.52 (14.12 to 29.14) | 0.44 (0.27 to 0.63) | 20.75 (13.56 to 28.05) | 0.28 (0.16 to 0.45) | -3.67% (-4.86 to -2.45) |  | 21.52 (14.14 to 29.15) | 41.6 (26.19 to 59.54) | 20.75 (13.56 to 27.95) | 27.16 (15.81 to 42.22) | -3.58% (-4.7 to -2.44) |
| Cuba | 10.9 (6.53 to 15.69) | 1.34 (0.81 to 1.99) | 10.31 (6.25 to 14.8) | 0.87 (0.49 to 1.35) | -4.59% (-5.41 to -3.76) |  | 10.9 (6.53 to 15.7) | 119.11 (72.04 to 177.13) | 10.31 (6.25 to 14.8) | 77.87 (43.42 to 120) | -4.57% (-5.38 to -3.75) |
| Cyprus | 18.66 (12.02 to 25.2) | 0.24 (0.15 to 0.33) | 18.38 (11.92 to 24.65) | 0.17 (0.1 to 0.27) | -2.88% (-3.82 to -1.93) |  | 18.66 (12.04 to 25.18) | 22.22 (14.03 to 31.03) | 18.38 (11.91 to 24.67) | 16.19 (9.53 to 24.86) | -2.78% (-3.67 to -1.87) |
| Czechia | 17.01 (10.92 to 22.98) | 0.64 (0.39 to 0.89) | 17.15 (11.07 to 23.48) | 0.4 (0.23 to 0.6) | -3.2% (-5.08 to -1.28) |  | 17.01 (10.91 to 22.98) | 57.57 (35.33 to 80.64) | 17.15 (11.07 to 23.5) | 36.69 (20.87 to 54.99) | -3.08% (-4.91 to -1.21) |
| C么te d'Ivoire | 7.2 (4 to 10.7) | 25.06 (12.43 to 40) | 6.83 (3.75 to 10.17) | 15.17 (7.06 to 25.63) | -5.37% (-6.24 to -4.49) |  | 7.2 (4 to 10.7) | 2,200.83 (1,095.44 to 3,514.57) | 6.83 (3.75 to 10.17) | 1,332.14 (619.9 to 2,249.93) | -5.37% (-6.23 to -4.51) |
| Democratic People's Republic of Korea | 17.63 (11.15 to 24) | 13.68 (7.8 to 20.63) | 18.02 (11.46 to 24.38) | 6.13 (3.4 to 9.63) | -8.56% (-8.66 to -8.45) |  | 17.63 (11.15 to 23.99) | 1,208.8 (690.03 to 1,822.73) | 18.02 (11.46 to 24.38) | 542.51 (301.49 to 850.98) | -8.54% (-8.64 to -8.44) |
| Democratic Republic of the Congo | 1.53 (0.5 to 2.98) | 3.78 (1.1 to 7.96) | 1.37 (0.39 to 2.82) | 1.41 (0.35 to 3.4) | -10.48% (-11.21 to -9.74) |  | 1.53 (0.5 to 2.98) | 332.08 (96.84 to 699.82) | 1.37 (0.39 to 2.82) | 124.55 (31.33 to 299.92) | -10.45% (-11.18 to -9.72) |
| Denmark | 15.44 (9.94 to 20.99) | 0.22 (0.13 to 0.31) | 14.53 (9.16 to 20.01) | 0.16 (0.09 to 0.26) | -2.8% (-3.55 to -2.04) |  | 15.44 (9.96 to 20.97) | 20.26 (12.4 to 29.19) | 14.53 (9.19 to 19.99) | 15.63 (8.97 to 23.99) | -2.7% (-3.36 to -2.05) |
| Djibouti | 8.66 (3.94 to 14.29) | 25.7 (10.49 to 46.17) | 8.33 (3.76 to 13.77) | 12.71 (5.09 to 23.02) | -7.63% (-7.82 to -7.44) |  | 8.66 (3.94 to 14.29) | 2,257.12 (922.59 to 4,060.29) | 8.33 (3.76 to 13.77) | 1,116.12 (446.88 to 2,020.54) | -7.63% (-7.82 to -7.45) |
| Dominica | 5.42 (2.87 to 8.25) | 1.64 (0.77 to 2.72) | 5.6 (2.97 to 8.61) | 2.12 (0.97 to 3.7) | 2.72% (2.35 to 3.1) |  | 5.42 (2.86 to 8.24) | 144.74 (68.39 to 239.53) | 5.6 (2.97 to 8.61) | 186.78 (85.65 to 326.88) | 2.72% (2.35 to 3.09) |
| Dominican Republic | 5.86 (3.34 to 8.66) | 2.96 (1.59 to 4.51) | 5.64 (3.24 to 8.34) | 1.43 (0.64 to 2.49) | -8.53% (-9.62 to -7.42) |  | 5.86 (3.34 to 8.66) | 261.58 (140.3 to 398.23) | 5.64 (3.24 to 8.34) | 126.28 (57.08 to 220.35) | -8.52% (-9.61 to -7.42) |
| Ecuador | 2.92 (1.09 to 5.5) | 2.25 (0.77 to 4.48) | 2.75 (0.96 to 5.24) | 1.02 (0.32 to 2.08) | -7.98% (-8.95 to -7.01) |  | 2.92 (1.09 to 5.5) | 198.18 (67.57 to 394.9) | 2.75 (0.96 to 5.24) | 89.87 (28.62 to 182.87) | -7.97% (-8.93 to -7) |
| Egypt | 17.31 (11.22 to 23.47) | 21.09 (12.14 to 32.04) | 16.24 (10.31 to 22.19) | 9.37 (4.85 to 15.39) | -8.88% (-9.76 to -7.98) |  | 17.31 (11.22 to 23.47) | 1,862.32 (1,073.87 to 2,831.31) | 16.24 (10.31 to 22.19) | 828.04 (429.59 to 1,361.67) | -8.87% (-9.75 to -7.98) |
| El Salvador | 3.28 (1.62 to 5.11) | 1.4 (0.66 to 2.37) | 3.41 (1.74 to 5.38) | 0.72 (0.31 to 1.34) | -7.78% (-8.48 to -7.07) |  | 3.28 (1.62 to 5.11) | 123.64 (58.93 to 209.56) | 3.41 (1.74 to 5.38) | 63.78 (27.56 to 118.88) | -7.73% (-8.42 to -7.03) |
| Equatorial Guinea | 3.91 (1.61 to 6.83) | 3.56 (1.32 to 7.12) | 3.94 (1.63 to 6.8) | 1.56 (0.49 to 3.25) | -9.34% (-9.82 to -8.86) |  | 3.91 (1.61 to 6.83) | 314 (116.86 to 627.77) | 3.94 (1.63 to 6.8) | 138.05 (43.87 to 287.49) | -9.32% (-9.8 to -8.84) |
| Eritrea | 3.67 (1.95 to 5.73) | 9.19 (4.19 to 15.99) | 3.95 (2.07 to 6.07) | 5.83 (2.79 to 10.17) | -4.89% (-5.22 to -4.56) |  | 3.67 (1.95 to 5.73) | 805.39 (366.7 to 1,401.5) | 3.95 (2.07 to 6.08) | 510.82 (244.43 to 892.75) | -4.89% (-5.22 to -4.56) |
| Estonia | 17.18 (11.01 to 23.49) | 1.37 (0.82 to 2) | 16.8 (10.75 to 23.08) | 0.76 (0.42 to 1.18) | -5.16% (-6.23 to -4.07) |  | 17.18 (11.01 to 23.49) | 122.16 (73.47 to 177.57) | 16.8 (10.75 to 23.1) | 68.44 (38.53 to 105.39) | -5.06% (-6.12 to -3.99) |
| Eswatini | 2.47 (0.95 to 4.49) | 7.24 (2.73 to 14.62) | 2.43 (0.93 to 4.4) | 4.09 (1.45 to 8.26) | -6.29% (-6.93 to -5.65) |  | 2.47 (0.95 to 4.49) | 639.44 (240.89 to 1,291.15) | 2.43 (0.93 to 4.4) | 360.79 (127.42 to 727.93) | -6.31% (-6.95 to -5.66) |
| Ethiopia | 1.36 (0.51 to 2.48) | 3.38 (1.2 to 6.43) | 1.35 (0.47 to 2.55) | 1.58 (0.53 to 3.18) | -7.85% (-8.23 to -7.47) |  | 1.36 (0.51 to 2.48) | 297.36 (105.84 to 565.73) | 1.35 (0.47 to 2.55) | 139.41 (47.05 to 279.54) | -7.84% (-8.22 to -7.46) |
| Fiji | 6.94 (3.05 to 11.67) | 4.67 (1.83 to 8.01) | 7.34 (3.36 to 12.31) | 3.76 (1.48 to 6.7) | -2.31% (-2.65 to -1.96) |  | 6.94 (3.05 to 11.68) | 410.07 (160.98 to 704.87) | 7.34 (3.36 to 12.32) | 330.37 (129.8 to 588.35) | -2.31% (-2.65 to -1.97) |
| Finland | 12.78 (7.94 to 17.54) | 0.07 (0.04 to 0.11) | 12.48 (7.79 to 17.46) | 0.05 (0.03 to 0.07) | -4.94% (-6.32 to -3.55) |  | 12.78 (7.96 to 17.6) | 7.66 (4.49 to 11.23) | 12.48 (7.77 to 17.34) | 5.39 (3.11 to 8.13) | -4.2% (-5.37 to -3.01) |
| France | 14.34 (8.48 to 20.32) | 0.16 (0.09 to 0.24) | 13.75 (8.08 to 19.68) | 0.12 (0.06 to 0.18) | -3.02% (-3.7 to -2.33) |  | 14.34 (8.48 to 20.33) | 15.02 (8.62 to 22.41) | 13.75 (8.09 to 19.67) | 11.15 (6.22 to 16.8) | -2.83% (-3.44 to -2.22) |
| Gabon | 4.87 (2.24 to 8.06) | 4.49 (1.81 to 8.49) | 4.62 (1.98 to 7.57) | 1.79 (0.64 to 3.57) | -9.56% (-10.53 to -8.59) |  | 4.87 (2.24 to 8.06) | 396.25 (160.42 to 748.27) | 4.62 (1.98 to 7.57) | 158.18 (56.94 to 315.9) | -9.55% (-10.52 to -8.58) |
| Gambia | 7.63 (4.03 to 11.52) | 11.74 (5.53 to 18.98) | 7.41 (3.89 to 11.33) | 6.49 (2.96 to 11.38) | -5.5% (-6.38 to -4.61) |  | 7.63 (4.03 to 11.52) | 1,030.49 (486.46 to 1,666.43) | 7.41 (3.89 to 11.34) | 570.27 (261.25 to 999.71) | -5.5% (-6.37 to -4.61) |
| Georgia | 21.86 (14.62 to 28.97) | 6.88 (4.25 to 9.92) | 22.47 (15.45 to 29.69) | 3.36 (1.91 to 5.15) | -7.43% (-9.18 to -5.66) |  | 21.86 (14.62 to 28.98) | 605.62 (374.35 to 872.57) | 22.47 (15.45 to 29.69) | 296.08 (168.58 to 453.72) | -7.42% (-9.16 to -5.64) |
| Germany | 15.58 (9.92 to 21.61) | 0.2 (0.13 to 0.29) | 15.01 (9.35 to 20.92) | 0.13 (0.08 to 0.2) | -4.33% (-5.32 to -3.34) |  | 15.58 (9.93 to 21.59) | 19.11 (12.05 to 27.35) | 15.01 (9.43 to 20.98) | 12.55 (7.47 to 18.83) | -4.13% (-5.06 to -3.19) |
| Ghana | 2.05 (0.96 to 3.41) | 2.84 (1.19 to 5.13) | 2.13 (1.02 to 3.48) | 1.94 (0.73 to 3.61) | -3.12% (-4.78 to -1.43) |  | 2.05 (0.96 to 3.41) | 249.97 (104.71 to 450.74) | 2.13 (1.02 to 3.48) | 170.52 (64.05 to 317.45) | -3.12% (-4.77 to -1.45) |
| Greece | 21.95 (14.63 to 29.23) | 0.76 (0.45 to 1.21) | 20.75 (13.58 to 27.86) | 0.57 (0.33 to 0.9) | -1.28% (-3.66 to 1.16) |  | 21.95 (14.63 to 29.21) | 69.17 (41.32 to 110.23) | 20.75 (13.58 to 27.85) | 51.55 (29.82 to 82.17) | -1.36% (-3.65 to 0.99) |
| Greenland | 15.29 (8.88 to 21.8) | 1.41 (0.77 to 2.14) | 15 (8.87 to 21.23) | 0.88 (0.41 to 1.54) | -4.87% (-5.49 to -4.25) |  | 15.29 (8.88 to 21.79) | 124.74 (68.28 to 189.89) | 15 (8.87 to 21.23) | 78.4 (37.31 to 136.66) | -4.81% (-5.42 to -4.2) |
| Grenada | 5.32 (2.51 to 8.55) | 2.11 (0.93 to 3.62) | 5.46 (2.7 to 8.68) | 1.72 (0.77 to 3.03) | -2.26% (-2.77 to -1.74) |  | 5.32 (2.51 to 8.55) | 186.13 (81.85 to 318.73) | 5.46 (2.7 to 8.68) | 151.69 (67.74 to 267.6) | -2.24% (-2.76 to -1.72) |
| Guam | 9.88 (5.09 to 15.04) | 2.88 (1.44 to 4.55) | 9.89 (5.21 to 14.8) | 2.7 (1.25 to 4.42) | -0.56% (-0.78 to -0.34) |  | 9.88 (5.09 to 15.04) | 254.94 (127.26 to 402.19) | 9.89 (5.21 to 14.8) | 238.7 (110.49 to 390.75) | -0.57% (-0.79 to -0.34) |
| Guatemala | 3.1 (1.2 to 5.64) | 6.08 (2.27 to 11.12) | 3.17 (1.25 to 5.68) | 3.81 (1.35 to 7.12) | -5.06% (-5.31 to -4.82) |  | 3.1 (1.2 to 5.64) | 534.3 (198.91 to 975.65) | 3.17 (1.26 to 5.68) | 335.4 (119.04 to 626.87) | -5.05% (-5.3 to -4.8) |
| Guinea | 4.9 (2.45 to 7.68) | 23.32 (11.11 to 40.52) | 5.05 (2.64 to 7.95) | 17.2 (7.85 to 29.44) | -2.71% (-3.5 to -1.9) |  | 4.9 (2.45 to 7.68) | 2,040.94 (971.15 to 3,534) | 5.05 (2.64 to 7.95) | 1,505.75 (688.52 to 2,576.68) | -2.7% (-3.49 to -1.91) |
| Guinea-Bissau | 4.68 (2.32 to 7.49) | 10.42 (4.83 to 18.43) | 4.77 (2.42 to 7.45) | 4.95 (2.16 to 9.16) | -7.07% (-8.17 to -5.97) |  | 4.68 (2.31 to 7.48) | 912.98 (423.5 to 1,616.09) | 4.77 (2.42 to 7.45) | 435 (189.58 to 803.31) | -7.05% (-8.14 to -5.95) |
| Guyana | 7.21 (3.78 to 11) | 3.85 (1.85 to 6.53) | 7.43 (3.95 to 11.27) | 2.74 (1.24 to 4.74) | -3.96% (-5.16 to -2.73) |  | 7.21 (3.77 to 11) | 339.99 (163.22 to 575.65) | 7.43 (3.95 to 11.27) | 241.76 (109.3 to 418.21) | -3.95% (-5.15 to -2.73) |
| Haiti | 2.74 (1.17 to 4.73) | 7.72 (3.01 to 14.14) | 3.2 (1.46 to 5.24) | 6.68 (2.93 to 11.78) | -1.56% (-2.1 to -1.03) |  | 2.74 (1.17 to 4.73) | 679.11 (265.25 to 1,243.78) | 3.2 (1.46 to 5.24) | 587.54 (257.49 to 1,034.99) | -1.56% (-2.09 to -1.03) |
| Honduras | 6.83 (3.65 to 10.22) | 2.52 (1.11 to 4.4) | 6.87 (3.7 to 10.31) | 1.45 (0.57 to 2.75) | -5.92% (-6.16 to -5.68) |  | 6.83 (3.65 to 10.21) | 223.34 (98.56 to 388.88) | 6.87 (3.7 to 10.31) | 128.55 (51.57 to 243.89) | -5.9% (-6.14 to -5.66) |
| Hungary | 19.86 (12.84 to 26.94) | 1.05 (0.64 to 1.49) | 19.43 (12.49 to 26.63) | 0.69 (0.39 to 1.08) | -4.7% (-6.51 to -2.86) |  | 19.86 (12.84 to 26.94) | 94.77 (57.37 to 134.22) | 19.43 (12.5 to 26.63) | 62.97 (36.12 to 98.35) | -4.6% (-6.36 to -2.8) |
| Iceland | 16.83 (11.18 to 22.89) | 0.28 (0.17 to 0.42) | 17.54 (11.87 to 24.01) | 0.24 (0.12 to 0.41) | -0.97% (-1.75 to -0.19) |  | 16.82 (11.17 to 22.88) | 25.95 (16.01 to 38.76) | 17.54 (11.88 to 24.02) | 22.22 (11.67 to 37.44) | -0.92% (-1.68 to -0.16) |
| India | 9.33 (5.06 to 13.86) | 18.76 (9.97 to 28.68) | 9.98 (5.54 to 14.79) | 10.95 (5.8 to 17.03) | -6.28% (-7.17 to -5.38) |  | 9.33 (5.06 to 13.87) | 1,655.47 (878.15 to 2,532.33) | 9.98 (5.54 to 14.79) | 967.43 (512.88 to 1,506.16) | -6.27% (-7.15 to -5.37) |
| Indonesia | 14.63 (8.27 to 21.22) | 11.84 (6.05 to 18.3) | 15.83 (9.19 to 22.58) | 7.23 (3.8 to 11.2) | -5.37% (-5.6 to -5.15) |  | 14.63 (8.27 to 21.21) | 1,045.14 (534.6 to 1,615.96) | 15.83 (9.19 to 22.58) | 638.3 (334.99 to 988.47) | -5.37% (-5.59 to -5.15) |
| Iran (Islamic Republic of) | 13.21 (8.29 to 18.36) | 4.63 (2.75 to 6.67) | 13.23 (8.23 to 18.45) | 1.08 (0.61 to 1.66) | -16.09% (-17.93 to -14.22) |  | 13.21 (8.3 to 18.36) | 409.22 (243.19 to 589.47) | 13.23 (8.23 to 18.46) | 96.49 (54.87 to 147.5) | -16.03% (-17.85 to -14.16) |
| Iraq | 16.26 (10.48 to 22.02) | 8.63 (5.11 to 12.79) | 15.8 (10.08 to 21.62) | 3.44 (1.92 to 5.51) | -9.46% (-9.75 to -9.16) |  | 16.26 (10.48 to 22.02) | 761.95 (451.19 to 1,129.45) | 15.8 (10.08 to 21.62) | 303.59 (168.52 to 485.95) | -9.46% (-9.76 to -9.17) |
| Ireland | 13.19 (7.97 to 18.55) | 0.22 (0.13 to 0.32) | 12.24 (7.24 to 17.54) | 0.11 (0.06 to 0.18) | -6.68% (-7.73 to -5.61) |  | 13.19 (7.97 to 18.53) | 20.4 (11.66 to 29.78) | 12.24 (7.21 to 17.52) | 10.99 (6.05 to 16.87) | -6.34% (-7.37 to -5.29) |
| Israel | 14.36 (8.86 to 19.91) | 0.31 (0.19 to 0.44) | 13.32 (7.96 to 18.66) | 0.19 (0.11 to 0.3) | -5.04% (-5.76 to -4.32) |  | 14.36 (8.86 to 19.9) | 27.92 (17.1 to 40.13) | 13.32 (7.95 to 18.65) | 17.49 (9.82 to 27.69) | -4.86% (-5.55 to -4.17) |
| Italy | 15.27 (9.71 to 20.84) | 0.23 (0.14 to 0.31) | 14.73 (9.26 to 20.32) | 0.14 (0.09 to 0.2) | -5.1% (-5.41 to -4.8) |  | 15.27 (9.72 to 20.84) | 21.35 (13.49 to 29.71) | 14.72 (9.24 to 20.31) | 13.85 (8.58 to 19.56) | -4.88% (-5.15 to -4.6) |
| Jamaica | 8.95 (5.21 to 13.01) | 1.08 (0.57 to 1.77) | 9 (5.23 to 13.02) | 0.93 (0.46 to 1.58) | -1.82% (-2.59 to -1.04) |  | 8.95 (5.21 to 13.01) | 96.43 (50.67 to 157.52) | 9 (5.23 to 13.02) | 82.69 (41.52 to 140.06) | -1.81% (-2.57 to -1.04) |
| Japan | 17.92 (11.65 to 24.62) | 0.64 (0.41 to 0.88) | 17.5 (11.48 to 23.79) | 0.41 (0.26 to 0.58) | -5.03% (-5.3 to -4.75) |  | 17.92 (11.66 to 24.61) | 57.54 (36.79 to 78.56) | 17.5 (11.48 to 23.79) | 37.29 (23.93 to 52.01) | -4.93% (-5.19 to -4.67) |
| Jordan | 17.65 (11.32 to 23.9) | 6.08 (3.72 to 8.85) | 16.24 (10.21 to 22.38) | 4.1 (2.32 to 6.35) | -3.73% (-4.37 to -3.09) |  | 17.65 (11.32 to 23.89) | 538.68 (330.51 to 783.19) | 16.24 (10.21 to 22.38) | 363.73 (206.02 to 562.59) | -3.73% (-4.37 to -3.09) |
| Kazakhstan | 17.05 (10.49 to 23.48) | 12.07 (6.97 to 17.99) | 16.49 (10.05 to 22.71) | 5.06 (2.74 to 7.92) | -9.3% (-10.61 to -7.98) |  | 17.05 (10.49 to 23.48) | 1,063.64 (614.17 to 1,583.78) | 16.49 (10.05 to 22.7) | 445.61 (241.44 to 697.93) | -9.3% (-10.6 to -7.98) |
| Kenya | 4.18 (1.8 to 7.13) | 7.1 (2.84 to 12.47) | 3.97 (1.7 to 6.84) | 3.9 (1.49 to 7.07) | -6.79% (-7.08 to -6.49) |  | 4.18 (1.8 to 7.13) | 625.88 (250.49 to 1,100.55) | 3.97 (1.7 to 6.84) | 343.92 (131.46 to 623.69) | -6.78% (-7.07 to -6.48) |
| Kiribati | 12.96 (7.09 to 19.16) | 10.99 (5.51 to 18.5) | 12.36 (6.67 to 18.58) | 5.93 (2.77 to 10.41) | -6.8% (-7.09 to -6.51) |  | 12.96 (7.09 to 19.16) | 964.42 (484.6 to 1,626.66) | 12.36 (6.67 to 18.58) | 521.31 (244.57 to 913.45) | -6.78% (-7.07 to -6.5) |
| Kuwait | 17.96 (11.57 to 24.47) | 2.61 (1.63 to 3.64) | 18.3 (12.26 to 24.58) | 1.87 (1.12 to 2.82) | -3.38% (-4.33 to -2.43) |  | 17.96 (11.57 to 24.47) | 231.33 (144.72 to 322.16) | 18.3 (12.26 to 24.58) | 166.25 (99.84 to 250.52) | -3.35% (-4.29 to -2.41) |
| Kyrgyzstan | 19.48 (12.73 to 26.2) | 20.74 (13.46 to 27.7) | 20.5 (13.54 to 27.54) | 11.16 (7.07 to 15.41) | -6.89% (-7.6 to -6.17) |  | 19.48 (12.73 to 26.2) | 1,824.56 (1,185.71 to 2,435.54) | 20.5 (13.53 to 27.54) | 980.19 (621.88 to 1,354.05) | -6.91% (-7.62 to -6.19) |
| Lao People's Democratic Republic | 13 (7.24 to 18.93) | 52.14 (26.71 to 85.02) | 13.02 (7.18 to 19.28) | 24.62 (11.98 to 40.58) | -8.13% (-8.46 to -7.8) |  | 13 (7.24 to 18.93) | 4,600.7 (2,354.07 to 7,504.59) | 13.02 (7.18 to 19.28) | 2,174.66 (1,059.26 to 3,581.51) | -8.12% (-8.45 to -7.79) |
| Latvia | 16.86 (10.37 to 23.36) | 1.57 (0.95 to 2.27) | 16.26 (9.77 to 22.84) | 0.83 (0.44 to 1.3) | -7.79% (-8.86 to -6.72) |  | 16.86 (10.37 to 23.36) | 140 (84.61 to 202.45) | 16.25 (9.78 to 22.84) | 74.45 (39.38 to 116.41) | -7.7% (-8.74 to -6.65) |
| Lebanon | 20.8 (14.09 to 28.01) | 3.1 (1.56 to 5.14) | 19.45 (12.91 to 26.4) | 1.52 (0.66 to 2.85) | -7.25% (-7.72 to -6.78) |  | 20.8 (14.09 to 28.01) | 276.06 (140.23 to 455.55) | 19.46 (12.92 to 26.4) | 135.96 (61.16 to 253.4) | -7.18% (-7.64 to -6.72) |
| Lesotho | 11.54 (6.96 to 16.36) | 36.89 (19.42 to 57.09) | 10.62 (6.2 to 15.49) | 25.6 (13.45 to 40.75) | -4.64% (-5.44 to -3.84) |  | 11.54 (6.96 to 16.36) | 3,253.73 (1,713.9 to 5,040.91) | 10.62 (6.2 to 15.48) | 2,256.19 (1,186.96 to 3,594.02) | -4.65% (-5.44 to -3.85) |
| Liberia | 2.68 (1.15 to 4.55) | 3.98 (1.45 to 7.63) | 2.63 (1.1 to 4.47) | 2.34 (0.89 to 4.66) | -6.35% (-7.29 to -5.4) |  | 2.68 (1.15 to 4.55) | 349.72 (128.11 to 670.39) | 2.63 (1.1 to 4.46) | 205.7 (78.1 to 409.89) | -6.33% (-7.26 to -5.39) |
| Libya | 16.79 (11.18 to 22.93) | 2.92 (1.7 to 4.51) | 16.78 (10.98 to 22.92) | 1.84 (1 to 2.95) | -4.73% (-5.55 to -3.9) |  | 16.79 (11.18 to 22.93) | 256.03 (150.02 to 393.94) | 16.78 (10.99 to 22.91) | 162.19 (88.61 to 260.09) | -4.69% (-5.52 to -3.86) |
| Lithuania | 15.42 (9.28 to 21.45) | 1.51 (0.92 to 2.17) | 15.47 (9.46 to 21.49) | 0.89 (0.5 to 1.35) | -4.87% (-6.31 to -3.41) |  | 15.41 (9.28 to 21.43) | 134.94 (81.9 to 194.03) | 15.47 (9.46 to 21.47) | 80.15 (44.94 to 121.44) | -4.82% (-6.24 to -3.39) |
| Luxembourg | 16.32 (10.52 to 22.37) | 0.21 (0.12 to 0.32) | 15.47 (9.97 to 21.29) | 0.15 (0.07 to 0.26) | -3.27% (-4.06 to -2.48) |  | 16.32 (10.52 to 22.37) | 19.89 (11.8 to 29.49) | 15.47 (9.94 to 21.28) | 14.07 (7.39 to 24.64) | -3.2% (-3.95 to -2.45) |
| Madagascar | 3.45 (1.06 to 6.81) | 8.4 (2.36 to 17.27) | 3.43 (1.09 to 6.69) | 4.98 (1.36 to 10.13) | -5.7% (-5.99 to -5.41) |  | 3.45 (1.06 to 6.81) | 739.68 (207.44 to 1,523.23) | 3.43 (1.09 to 6.69) | 439.6 (120.2 to 894.25) | -5.68% (-5.97 to -5.39) |
| Malawi | 3.26 (1.3 to 5.84) | 8.42 (3.17 to 15.98) | 3.34 (1.39 to 5.86) | 5.3 (2.03 to 10.21) | -5.41% (-5.88 to -4.93) |  | 3.26 (1.3 to 5.84) | 739.86 (278.35 to 1,404.41) | 3.34 (1.39 to 5.86) | 465.97 (178.06 to 897) | -5.4% (-5.87 to -4.93) |
| Malaysia | 13.53 (7.8 to 19.34) | 1.27 (0.69 to 1.93) | 13.03 (7.39 to 18.96) | 0.98 (0.49 to 1.67) | -2.38% (-4.71 to 0.02) |  | 13.53 (7.8 to 19.34) | 113.2 (61.51 to 171.89) | 13.03 (7.4 to 18.96) | 88.05 (44 to 148.92) | -2.33% (-4.63 to 0.04) |
| Maldives | 15.99 (9.53 to 22.55) | 2.13 (1.15 to 3.29) | 15.59 (9.32 to 21.9) | 1.47 (0.73 to 2.42) | -3.53% (-4.23 to -2.84) |  | 15.99 (9.53 to 22.55) | 188.55 (102.38 to 290.89) | 15.59 (9.31 to 21.9) | 131.03 (65.24 to 214.81) | -3.5% (-4.19 to -2.81) |
| Mali | 3.66 (1.58 to 6.22) | 13.11 (5.2 to 24.24) | 3.86 (1.71 to 6.46) | 11.12 (4.48 to 20.2) | -1.08% (-1.84 to -0.32) |  | 3.66 (1.58 to 6.23) | 1,147.69 (455.32 to 2,123.17) | 3.86 (1.71 to 6.46) | 974.47 (392.84 to 1,769.03) | -1.08% (-1.83 to -0.32) |
| Malta | 17.78 (11.94 to 24.27) | 0.74 (0.46 to 1.07) | 16.92 (11.27 to 23.13) | 0.55 (0.29 to 0.91) | -2.74% (-3.34 to -2.14) |  | 17.78 (11.93 to 24.28) | 67.21 (41.67 to 96.49) | 16.92 (11.27 to 23.1) | 50.21 (26.94 to 81.8) | -2.72% (-3.3 to -2.13) |
| Marshall Islands | 9.41 (4.79 to 14.41) | 10.23 (4.88 to 16.87) | 9.99 (5.22 to 15.15) | 6.42 (3.12 to 10.61) | -5.08% (-5.54 to -4.61) |  | 9.41 (4.79 to 14.42) | 899.84 (429.24 to 1,485.63) | 9.99 (5.21 to 15.15) | 565.24 (274.52 to 932.31) | -5.06% (-5.53 to -4.6) |
| Mauritania | 4.44 (2.05 to 7.19) | 7.63 (3.25 to 13.66) | 4.12 (1.86 to 6.75) | 3.63 (1.42 to 6.94) | -8.4% (-9.18 to -7.62) |  | 4.44 (2.04 to 7.17) | 672.88 (287.42 to 1,206.99) | 4.12 (1.86 to 6.75) | 320.54 (125.59 to 613.12) | -8.39% (-9.16 to -7.6) |
| Mauritius | 11.35 (6.21 to 16.83) | 1.69 (0.86 to 2.65) | 11.98 (6.71 to 17.75) | 1.51 (0.76 to 2.49) | -1.89% (-3.06 to -0.69) |  | 11.35 (6.21 to 16.83) | 149.54 (75.93 to 233.76) | 11.98 (6.71 to 17.75) | 133.32 (67.81 to 220.12) | -1.87% (-3.04 to -0.68) |
| Mexico | 3.82 (1.17 to 7.53) | 1.63 (0.49 to 3.32) | 3.34 (0.91 to 6.79) | 0.86 (0.22 to 1.84) | -7.32% (-7.87 to -6.78) |  | 3.82 (1.17 to 7.53) | 144.29 (43.12 to 293.72) | 3.34 (0.91 to 6.79) | 76.46 (19.83 to 162.76) | -7.32% (-7.86 to -6.77) |
| Micronesia (Federated States of) | 8.12 (3.72 to 13.49) | 4.62 (1.84 to 8.38) | 8.09 (3.67 to 13.51) | 3.07 (1.2 to 5.49) | -4.37% (-4.64 to -4.1) |  | 8.12 (3.72 to 13.49) | 407.32 (162.3 to 738.2) | 8.09 (3.67 to 13.52) | 270.13 (106.35 to 483.94) | -4.38% (-4.64 to -4.11) |
| Monaco | 17.02 (10.99 to 23.02) | 0.44 (0.24 to 0.72) | 15.99 (10.12 to 22.08) | 0.26 (0.14 to 0.42) | -5.73% (-5.98 to -5.48) |  | 17.02 (10.98 to 23.06) | 39.78 (22.04 to 64.42) | 15.98 (10.13 to 22.06) | 24.32 (13.45 to 38.22) | -5.56% (-5.81 to -5.31) |
| Mongolia | 13.26 (7.59 to 19.33) | 24.03 (13.21 to 36.5) | 14.69 (8.71 to 20.86) | 10.65 (5.58 to 16.67) | -9.24% (-10.3 to -8.17) |  | 13.26 (7.59 to 19.33) | 2,119.05 (1,164.87 to 3,213.14) | 14.69 (8.71 to 20.86) | 939.21 (491.94 to 1,469.29) | -9.24% (-10.29 to -8.18) |
| Montenegro | 23.52 (15.97 to 31) | 2.33 (1.5 to 3.28) | 23.07 (15.71 to 30.59) | 0.9 (0.52 to 1.42) | -9.86% (-11.16 to -8.54) |  | 23.52 (15.97 to 31) | 208.17 (133.96 to 292.03) | 23.07 (15.7 to 30.59) | 82.01 (47.59 to 127.68) | -9.69% (-10.99 to -8.38) |
| Morocco | 11.09 (6.67 to 15.56) | 8.2 (3.94 to 13.79) | 10.66 (6.21 to 15.04) | 3.32 (1.45 to 6.24) | -9.34% (-9.59 to -9.09) |  | 11.09 (6.67 to 15.56) | 724.88 (349.85 to 1220.77) | 10.66 (6.21 to 15.04) | 294.41 (128.07 to 551.74) | -9.32% (-9.56 to -9.08) |
| Mozambique | 3.71 (1.17 to 7.19) | 9.02 (2.81 to 17.86) | 3.64 (1.2 to 6.88) | 5.26 (1.63 to 10.98) | -6.39% (-7.18 to -5.58) |  | 3.71 (1.17 to 7.19) | 794.74 (248.04 to 1,573.14) | 3.64 (1.2 to 6.88) | 463.81 (143.44 to 968.35) | -6.37% (-7.16 to -5.57) |
| Myanmar | 8.98 (4.15 to 14.6) | 26.1 (11.51 to 46.71) | 8.5 (3.94 to 14.07) | 11.19 (4.4 to 20.78) | -8.91% (-9.16 to -8.66) |  | 8.98 (4.15 to 14.6) | 2,304.57 (1,015.72 to 4,120.93) | 8.5 (3.94 to 14.07) | 988.78 (390 to 1,838.13) | -8.9% (-9.15 to -8.65) |
| Namibia | 4.58 (1.77 to 8.35) | 6.33 (2.18 to 12.23) | 4.52 (1.73 to 8.19) | 3.97 (1.27 to 8.28) | -4.84% (-5.27 to -4.42) |  | 4.58 (1.77 to 8.35) | 557.96 (192.13 to 1,077.76) | 4.52 (1.73 to 8.2) | 350.44 (112.14 to 729.61) | -4.84% (-5.27 to -4.41) |
| Nauru | 8.6 (3.64 to 14.54) | 18.23 (7.19 to 33.74) | 8.74 (3.93 to 14.6) | 8.42 (3.39 to 15) | -8.67% (-9.41 to -7.92) |  | 8.6 (3.65 to 14.55) | 1,595.21 (630.02 to 2,959.89) | 8.74 (3.93 to 14.6) | 737.47 (297.2 to 1,316.65) | -8.65% (-9.39 to -7.91) |
| Nepal | 6.41 (2.61 to 11.26) | 16.28 (6.29 to 28.9) | 7.24 (3.21 to 12.2) | 8.3 (3.12 to 14.61) | -6.9% (-7.38 to -6.42) |  | 6.41 (2.61 to 11.27) | 1,437.76 (556.44 to 2,550.62) | 7.24 (3.21 to 12.2) | 734.38 (275.63 to 1,291.82) | -6.89% (-7.36 to -6.41) |
| Netherlands | 13.25 (7.98 to 18.9) | 0.2 (0.12 to 0.29) | 13.26 (8.27 to 18.76) | 0.13 (0.07 to 0.2) | -4.53% (-5.76 to -3.29) |  | 13.25 (7.98 to 18.89) | 18.45 (11.03 to 27.18) | 13.26 (8.27 to 18.78) | 12.18 (6.97 to 18.71) | -4.27% (-5.45 to -3.08) |
| New Zealand | 11.31 (6.41 to 16.58) | 0.46 (0.26 to 0.69) | 11.63 (6.71 to 16.98) | 0.3 (0.17 to 0.47) | -4.97% (-6.24 to -3.69) |  | 11.31 (6.41 to 16.57) | 42.1 (23.34 to 63.48) | 11.63 (6.71 to 16.94) | 27.81 (15.29 to 43.68) | -4.82% (-6.02 to -3.6) |
| Nicaragua | 5.43 (2.59 to 8.81) | 4.52 (2.07 to 7.51) | 5.52 (2.49 to 9) | 2.76 (1.19 to 4.7) | -5.17% (-5.46 to -4.88) |  | 5.43 (2.59 to 8.81) | 398.56 (182.61 to 662.25) | 5.52 (2.5 to 9.01) | 243 (104.57 to 413.89) | -5.17% (-5.45 to -4.88) |
| Niger | 3.15 (1.48 to 5.18) | 13.74 (5.93 to 25.08) | 3.73 (1.8 to 5.91) | 13.36 (6.05 to 23.58) | 0.06% (-1.17 to 1.3) |  | 3.15 (1.48 to 5.18) | 1,199.9 (519.12 to 2,184.83) | 3.73 (1.8 to 5.9) | 1,168.25 (529.68 to 2,058.65) | 0.07% (-1.16 to 1.3) |
| Nigeria | 2.6 (1.39 to 4) | 13.73 (6.78 to 22.21) | 2.75 (1.49 to 4.16) | 10.6 (5.44 to 17.09) | -2.33% (-3.03 to -1.62) |  | 2.6 (1.39 to 4) | 1,204.13 (594.22 to 1,947.76) | 2.75 (1.49 to 4.16) | 930.51 (478.24 to 1,501.37) | -2.32% (-3.02 to -1.61) |
| Niue | 12.83 (7.24 to 18.65) | 9.83 (4.89 to 16.26) | 12.72 (7.26 to 18.74) | 7.56 (3.71 to 12.68) | -2.96% (-3.12 to -2.79) |  | 12.83 (7.24 to 18.65) | 861.22 (429.65 to 1,423.62) | 12.72 (7.26 to 18.72) | 662.47 (324.49 to 1,111.21) | -2.96% (-3.12 to -2.79) |
| North Macedonia | 23.02 (15.19 to 30.55) | 2.64 (1.62 to 3.7) | 22.79 (14.97 to 30.36) | 2.06 (1.24 to 3.06) | -2.11% (-5.15 to 1.03) |  | 23.02 (15.19 to 30.56) | 235.39 (145.02 to 328.33) | 22.79 (14.97 to 30.35) | 184.43 (109.73 to 273.53) | -2.09% (-5.09 to 1.01) |
| Northern Mariana Islands | 9.66 (5.08 to 14.81) | 1.84 (0.87 to 3.06) | 10.01 (5.42 to 15.28) | 2.16 (1.08 to 3.74) | 2.79% (1.16 to 4.45) |  | 9.66 (5.08 to 14.8) | 161.26 (76.51 to 268.04) | 10.01 (5.42 to 15.27) | 189.99 (95.12 to 328.62) | 2.81% (1.19 to 4.46) |
| Norway | 13.31 (8.23 to 18.67) | 0.1 (0.06 to 0.15) | 13.07 (7.83 to 18.59) | 0.07 (0.04 to 0.1) | -3.55% (-4.03 to -3.06) |  | 13.3 (8.23 to 18.65) | 10.33 (6.23 to 14.94) | 13.06 (7.84 to 18.62) | 7.37 (4.39 to 10.9) | -3.13% (-3.58 to -2.67) |
| Oman | 11.14 (6.97 to 15.86) | 1.35 (0.79 to 2.04) | 11.75 (7.47 to 16.32) | 1.12 (0.6 to 1.71) | -1.72% (-3.27 to -0.13) |  | 11.14 (6.96 to 15.86) | 119.99 (69.6 to 180.58) | 11.75 (7.47 to 16.32) | 99.11 (53.72 to 151.86) | -1.69% (-3.24 to -0.12) |
| Pakistan | 9.32 (5.23 to 13.63) | 19.05 (10.2 to 29.07) | 8.73 (4.78 to 13.14) | 13.31 (6.82 to 21.53) | -3.58% (-4.17 to -3) |  | 9.32 (5.23 to 13.63) | 1,677.33 (898.49 to 2,556.86) | 8.73 (4.78 to 13.15) | 1,171.83 (599.6 to 1,896.38) | -3.59% (-4.17 to -3) |
| Palau | 12.44 (6.92 to 18.07) | 15.17 (7.64 to 24.22) | 12.29 (6.78 to 18.38) | 10.54 (5.22 to 17.44) | -4.05% (-4.25 to -3.85) |  | 12.44 (6.92 to 18.07) | 1,330.54 (670.81 to 2,124.78) | 12.29 (6.77 to 18.37) | 923.74 (457.92 to 1,526.21) | -4.06% (-4.26 to -3.86) |
| Palestine | 16.67 (10.59 to 22.81) | 3.08 (1.9 to 4.61) | 16.22 (10.47 to 22.29) | 1.29 (0.7 to 1.97) | -9.11% (-9.94 to -8.27) |  | 16.67 (10.59 to 22.82) | 272.72 (167.66 to 408.39) | 16.22 (10.47 to 22.29) | 115.26 (62.63 to 174.82) | -9.04% (-9.88 to -8.2) |
| Panama | 4.13 (2.27 to 6.19) | 2.13 (1.12 to 3.39) | 4.25 (2.37 to 6.36) | 1.56 (0.79 to 2.57) | -3.82% (-4.47 to -3.17) |  | 4.13 (2.27 to 6.19) | 187.29 (98.35 to 297.76) | 4.25 (2.37 to 6.35) | 137.28 (69.95 to 226.17) | -3.82% (-4.47 to -3.16) |
| Papua New Guinea | 9.73 (4.55 to 15.71) | 43.44 (18.21 to 75.87) | 9.74 (4.61 to 15.62) | 32.24 (13.89 to 54.51) | -3.25% (-3.37 to -3.13) |  | 9.73 (4.55 to 15.7) | 3,819.89 (1,600.02 to 6,668.15) | 9.74 (4.61 to 15.62) | 2,836.02 (1,222.82 to 4,795.64) | -3.25% (-3.36 to -3.13) |
| Paraguay | 7.9 (4.27 to 11.8) | 2.85 (1.45 to 4.5) | 8.49 (4.71 to 12.58) | 1.91 (0.89 to 3.23) | -5.12% (-6.25 to -3.98) |  | 7.9 (4.27 to 11.8) | 252.7 (128.5 to 398.09) | 8.49 (4.71 to 12.59) | 169.82 (78.81 to 286.8) | -5.09% (-6.21 to -3.95) |
| Peru | 2.91 (1.05 to 5.44) | 2.19 (0.72 to 4.22) | 3.13 (1.17 to 5.71) | 1.12 (0.36 to 2.25) | -6.91% (-7.49 to -6.33) |  | 2.91 (1.05 to 5.44) | 192.82 (63.74 to 371.96) | 3.13 (1.17 to 5.71) | 98.82 (31.72 to 199.11) | -6.9% (-7.47 to -6.32) |
| Philippines | 13.46 (7.74 to 19.49) | 14.74 (8.17 to 22.24) | 13.33 (7.65 to 19.31) | 10.12 (5.49 to 15.1) | -3.93% (-4.56 to -3.3) |  | 13.46 (7.74 to 19.49) | 1,297.3 (719.42 to 1,957.14) | 13.33 (7.65 to 19.31) | 890.55 (483.64 to 1,328.02) | -3.93% (-4.55 to -3.31) |
| Poland | 16.87 (10.43 to 23.19) | 1.06 (0.66 to 1.51) | 16.11 (10 to 22.39) | 0.55 (0.3 to 0.86) | -6.62% (-7.09 to -6.14) |  | 16.87 (10.43 to 23.2) | 94.52 (59.04 to 134.39) | 16.11 (10 to 22.39) | 49.23 (27.23 to 77.04) | -6.54% (-7.01 to -6.07) |
| Portugal | 13.47 (8.11 to 18.99) | 0.43 (0.26 to 0.63) | 12.86 (7.76 to 18.02) | 0.28 (0.15 to 0.44) | -4.29% (-5.85 to -2.7) |  | 13.47 (8.11 to 18.99) | 39.64 (23.71 to 57.44) | 12.86 (7.76 to 18.03) | 25.7 (14.33 to 39.72) | -4.17% (-5.66 to -2.66) |
| Puerto Rico | 7.88 (4.63 to 11.33) | 0.63 (0.36 to 0.94) | 8.29 (4.88 to 11.78) | 0.54 (0.28 to 0.86) | -2.87% (-4.84 to -0.85) |  | 7.88 (4.63 to 11.33) | 56.35 (31.94 to 84.04) | 8.29 (4.88 to 11.78) | 48.04 (25.36 to 76.69) | -2.84% (-4.8 to -0.83) |
| Qatar | 14.82 (9.55 to 20.51) | 0.96 (0.57 to 1.47) | 14.71 (9.64 to 20.23) | 0.5 (0.28 to 0.8) | -7.11% (-7.64 to -6.59) |  | 14.82 (9.55 to 20.54) | 86.42 (51.11 to 131.33) | 14.71 (9.61 to 20.23) | 45.84 (25.86 to 72.48) | -6.96% (-7.46 to -6.46) |
| Republic of Korea | 18.01 (11.59 to 24.43) | 0.36 (0.23 to 0.51) | 16.87 (10.6 to 23.34) | 0.2 (0.12 to 0.29) | -6.7% (-7.49 to -5.91) |  | 18.01 (11.59 to 24.43) | 32.53 (20.44 to 45.71) | 16.87 (10.59 to 23.35) | 18.28 (10.72 to 26.86) | -6.49% (-7.26 to -5.7) |
| Republic of Moldova | 19.33 (12.97 to 25.91) | 11.79 (7.64 to 16.39) | 19.27 (13.1 to 25.88) | 7.28 (4.46 to 10.74) | -4.99% (-5.25 to -4.72) |  | 19.33 (12.97 to 25.91) | 1,044.96 (676.95 to 1,452.29) | 19.27 (13.1 to 25.89) | 644.67 (394.42 to 950.96) | -5% (-5.27 to -4.73) |
| Romania | 18.66 (11.81 to 25.28) | 12.07 (7.63 to 16.58) | 17.18 (10.51 to 23.83) | 6.66 (3.85 to 9.87) | -6.34% (-6.7 to -5.97) |  | 18.66 (11.81 to 25.28) | 1,067.34 (674.4 to 1,467.23) | 17.18 (10.51 to 23.82) | 588.61 (340.55 to 871.75) | -6.33% (-6.69 to -5.97) |
| Russian Federation | 21.45 (14.19 to 28.62) | 4.47 (2.89 to 6.06) | 21.2 (14.02 to 28.22) | 2.45 (1.54 to 3.44) | -8.63% (-10.96 to -6.24) |  | 21.45 (14.19 to 28.63) | 395.77 (256.01 to 537.09) | 21.2 (14.02 to 28.22) | 217.49 (136.22 to 305.17) | -8.59% (-10.91 to -6.21) |
| Rwanda | 3.93 (1.94 to 6.26) | 9.76 (4.48 to 16.45) | 4.14 (2.05 to 6.49) | 5.21 (2.25 to 9.28) | -6.51% (-7.35 to -5.67) |  | 3.93 (1.94 to 6.26) | 856.17 (392.77 to 1,442.24) | 4.14 (2.05 to 6.49) | 457.95 (197.08 to 814.87) | -6.5% (-7.34 to -5.66) |
| Saint Kitts and Nevis | 7.15 (3.85 to 10.83) | 1.78 (0.89 to 2.95) | 7.12 (3.89 to 10.74) | 1.47 (0.71 to 2.47) | -1.64% (-2.42 to -0.85) |  | 7.15 (3.85 to 10.83) | 157.41 (78.71 to 259.58) | 7.12 (3.89 to 10.73) | 130.31 (62.36 to 218.13) | -1.62% (-2.4 to -0.83) |
| Saint Lucia | 5.72 (3.09 to 8.72) | 1.16 (0.57 to 1.92) | 5.83 (3.09 to 8.79) | 0.94 (0.44 to 1.66) | -2.56% (-2.85 to -2.26) |  | 5.72 (3.09 to 8.72) | 102.67 (50.21 to 169.5) | 5.83 (3.09 to 8.79) | 83.5 (38.85 to 147.08) | -2.55% (-2.85 to -2.24) |
| Saint Vincent and the Grenadines | 6.31 (3.22 to 9.66) | 1.69 (0.79 to 2.87) | 6.42 (3.43 to 9.83) | 1.32 (0.6 to 2.26) | -2.71% (-3.07 to -2.36) |  | 6.31 (3.22 to 9.67) | 149.46 (70.1 to 252.77) | 6.42 (3.43 to 9.83) | 116.65 (53.14 to 199.87) | -2.7% (-3.06 to -2.35) |
| Samoa | 15.32 (9.45 to 21.18) | 5.89 (3.07 to 9.44) | 14.73 (8.76 to 20.68) | 3.65 (1.76 to 6.04) | -4.39% (-5.48 to -3.29) |  | 15.32 (9.45 to 21.17) | 518.69 (272.3 to 832.2) | 14.73 (8.76 to 20.68) | 322.7 (156.37 to 533.71) | -4.38% (-5.45 to -3.29) |
| San Marino | 17.47 (11.5 to 24.28) | 0.22 (0.13 to 0.37) | 17.24 (11.38 to 23.81) | 0.18 (0.1 to 0.3) | -2.1% (-2.22 to -1.98) |  | 17.47 (11.53 to 24.28) | 20.95 (12.49 to 34.58) | 17.24 (11.38 to 23.82) | 17.42 (10.05 to 27.46) | -1.99% (-2.1 to -1.89) |
| Sao Tome and Principe | 1.34 (0.65 to 2.18) | 1.48 (0.64 to 2.62) | 1.33 (0.64 to 2.14) | 0.82 (0.34 to 1.48) | -4.09% (-6.44 to -1.67) |  | 1.34 (0.65 to 2.17) | 130.6 (56.47 to 230.91) | 1.33 (0.64 to 2.14) | 72.56 (30.23 to 130.15) | -4.09% (-6.43 to -1.68) |
| Saudi Arabia | 15.87 (10.33 to 22.24) | 0.78 (0.44 to 1.21) | 16.04 (10.45 to 22.11) | 0.27 (0.14 to 0.45) | -11.04% (-11.18 to -10.91) |  | 15.87 (10.33 to 22.25) | 69.27 (39.55 to 107.6) | 16.04 (10.44 to 22.01) | 25.26 (13.34 to 41.19) | -10.66% (-10.78 to -10.55) |
| Senegal | 7.48 (4.1 to 11.25) | 13.59 (6.92 to 22.34) | 7.61 (4.39 to 11.15) | 8.34 (4.03 to 14.24) | -4.24% (-5.54 to -2.93) |  | 7.48 (4.1 to 11.25) | 1,196.07 (609.75 to 1,965.17) | 7.61 (4.39 to 11.15) | 735.44 (356.71 to 1,255.99) | -4.23% (-5.52 to -2.93) |
| Serbia | 21.3 (13.95 to 28.38) | 1.19 (0.76 to 1.65) | 19.08 (12.2 to 25.89) | 0.53 (0.3 to 0.83) | -8.12% (-11.63 to -4.48) |  | 21.3 (13.95 to 28.38) | 107.35 (69.01 to 148.28) | 19.08 (12.19 to 25.87) | 48.55 (27.74 to 74.49) | -8% (-11.41 to -4.46) |
| Seychelles | 12.26 (7.08 to 17.45) | 3.86 (2.14 to 5.81) | 11.55 (6.54 to 16.66) | 3.02 (1.55 to 4.84) | -2.8% (-3.9 to -1.68) |  | 12.26 (7.07 to 17.44) | 341.2 (189.14 to 512.65) | 11.54 (6.54 to 16.66) | 267.31 (137.36 to 427.54) | -2.78% (-3.88 to -1.66) |
| Sierra Leone | 5.56 (2.06 to 10.02) | 31.76 (11.45 to 62.14) | 5.53 (2.15 to 9.84) | 18.91 (6.69 to 37.48) | -5.72% (-5.99 to -5.44) |  | 5.56 (2.06 to 10.02) | 2,789.5 (1,006.06 to 5451.13) | 5.53 (2.15 to 9.84) | 1,661.91 (588.39 to 3,289.07) | -5.71% (-5.99 to -5.43) |
| Singapore | 10.88 (6.43 to 15.61) | 0.77 (0.43 to 1.13) | 10.96 (6.44 to 15.63) | 0.38 (0.19 to 0.6) | -8.01% (-9.04 to -6.97) |  | 10.88 (6.43 to 15.61) | 68.55 (38.61 to 100.72) | 10.96 (6.44 to 15.63) | 34.49 (17.26 to 53.94) | -7.91% (-8.94 to -6.88) |
| Slovakia | 14.69 (9.01 to 20.44) | 1.94 (1.15 to 2.82) | 14.84 (9.14 to 20.87) | 1.28 (0.71 to 1.99) | -3.44% (-4.42 to -2.46) |  | 14.69 (9.02 to 20.44) | 173.06 (103.04 to 250.88) | 14.84 (9.14 to 20.86) | 114.52 (63.61 to 176.42) | -3.41% (-4.39 to -2.43) |
| Slovenia | 18.34 (12.17 to 24.74) | 0.37 (0.22 to 0.54) | 18.32 (12.09 to 24.58) | 0.15 (0.09 to 0.24) | -9.07% (-10.31 to -7.8) |  | 18.34 (12.14 to 24.71) | 34.5 (21.37 to 50.59) | 18.32 (12.15 to 24.57) | 15.68 (9.31 to 23.79) | -8.27% (-9.5 to -7.02) |
| Solomon Islands | 10.14 (5.13 to 15.41) | 15.66 (7.34 to 26.61) | 10.32 (5.31 to 15.7) | 9.89 (4.72 to 16.32) | -4.82% (-5.16 to -4.47) |  | 10.14 (5.13 to 15.41) | 1,378.91 (646.23 to 2,342.36) | 10.32 (5.31 to 15.71) | 871.07 (415.68 to 1,435.78) | -4.82% (-5.16 to -4.48) |
| Somalia | 3.59 (1.48 to 6.25) | 20.35 (7.26 to 38.91) | 3.45 (1.45 to 6.2) | 13.14 (4.97 to 25.79) | -5.24% (-5.69 to -4.79) |  | 3.59 (1.48 to 6.25) | 1,786.29 (637.66 to 3,414.4) | 3.45 (1.46 to 6.2) | 1,154.6 (436.74 to 2,270.06) | -5.23% (-5.68 to -4.78) |
| South Africa | 7.91 (3.79 to 12.48) | 9.86 (4.47 to 16.86) | 7.43 (3.56 to 12.08) | 5.76 (2.57 to 9.86) | -5.75% (-6.08 to -5.43) |  | 7.91 (3.79 to 12.48) | 872.36 (395.51 to 1,491.71) | 7.43 (3.56 to 12.08) | 508.22 (226.8 to 870.44) | -5.78% (-6.11 to -5.45) |
| South Sudan | 3.85 (1.68 to 6.54) | 18.2 (7.19 to 32.6) | 3.67 (1.55 to 6.3) | 13.03 (4.98 to 24.7) | -2.99% (-4.25 to -1.71) |  | 3.85 (1.68 to 6.54) | 1,599.11 (632.88 to 2,860.17) | 3.67 (1.55 to 6.3) | 1,145.23 (436.92 to 2,168.7) | -3% (-4.25 to -1.73) |
| Spain | 16.47 (10.29 to 22.72) | 0.29 (0.17 to 0.41) | 16.3 (10.23 to 22.24) | 0.18 (0.1 to 0.26) | -4.88% (-5.89 to -3.86) |  | 16.47 (10.27 to 22.74) | 26.37 (16.04 to 37.91) | 16.29 (10.23 to 22.29) | 16.61 (9.63 to 24.72) | -4.61% (-5.58 to -3.63) |
| Sri Lanka | 6.41 (2.56 to 11.28) | 0.99 (0.39 to 1.73) | 6.52 (2.66 to 11.31) | 0.5 (0.18 to 0.95) | -7.46% (-7.94 to -6.98) |  | 6.41 (2.56 to 11.28) | 87.26 (34.86 to 153.28) | 6.52 (2.66 to 11.31) | 44.2 (15.84 to 84.27) | -7.42% (-7.88 to -6.95) |
| Sudan | 11.91 (8.13 to 16.25) | 13.84 (6.75 to 26.31) | 12.39 (8.44 to 16.94) | 6.53 (3.25 to 11.83) | -7.89% (-8.38 to -7.39) |  | 11.91 (8.12 to 16.25) | 1,214.16 (592.44 to 2,310.4) | 12.39 (8.44 to 16.94) | 574.71 (286.23 to 1,040.08) | -7.87% (-8.36 to -7.37) |
| Suriname | 12.41 (7.35 to 17.68) | 6.21 (3.36 to 9.51) | 11.72 (6.75 to 16.89) | 3.77 (1.85 to 6.27) | -5.51% (-6.03 to -4.98) |  | 12.41 (7.35 to 17.68) | 548.91 (296.63 to 840.11) | 11.72 (6.75 to 16.89) | 333.18 (163.52 to 554.57) | -5.5% (-6.02 to -4.97) |
| Sweden | 9.95 (6.44 to 13.98) | 0.12 (0.07 to 0.17) | 10.3 (6.63 to 14.78) | 0.09 (0.05 to 0.14) | -2.06% (-3.1 to -1.01) |  | 9.96 (6.47 to 14.03) | 11.66 (7.19 to 17.27) | 10.3 (6.63 to 14.76) | 9.25 (5.35 to 14.08) | -2.1% (-3.01 to -1.18) |
| Switzerland | 14.47 (8.84 to 20.26) | 0.2 (0.12 to 0.31) | 13.98 (8.44 to 19.5) | 0.14 (0.08 to 0.22) | -3.79% (-4.63 to -2.94) |  | 14.47 (8.83 to 20.23) | 18.81 (10.69 to 28.36) | 13.98 (8.44 to 19.56) | 13.29 (7.71 to 20.08) | -3.59% (-4.39 to -2.79) |
| Syrian Arab Republic | 13.4 (8.04 to 18.75) | 4.35 (2.53 to 6.48) | 14.35 (8.78 to 20) | 3.4 (1.91 to 5.38) | -3.4% (-6.77 to 0.1) |  | 13.4 (8.04 to 18.76) | 378.94 (220.71 to 563.74) | 14.35 (8.78 to 20.02) | 297.02 (167.21 to 468.18) | -3.38% (-6.72 to 0.09) |
| Taiwan (Province of China) | 14.92 (9.39 to 20.59) | 0.69 (0.42 to 0.98) | 14.46 (8.87 to 20.24) | 0.55 (0.33 to 0.82) | -3.56% (-5.03 to -2.07) |  | 14.92 (9.39 to 20.59) | 61.43 (37.49 to 87.92) | 14.46 (8.9 to 20.25) | 49.81 (30 to 74.48) | -3.41% (-4.85 to -1.95) |
| Tajikistan | 12.52 (8 to 17.53) | 33.28 (20.13 to 47.52) | 12.39 (7.81 to 17.14) | 20.18 (11.48 to 32.21) | -5.41% (-5.61 to -5.21) |  | 12.52 (8.01 to 17.53) | 2,927.02 (1,768.34 to 4,175.98) | 12.39 (7.81 to 17.15) | 1,774.4 (1,009.5 to 2,832.66) | -5.41% (-5.62 to -5.21) |
| Thailand | 10.22 (5.6 to 15.12) | 1.79 (0.91 to 2.69) | 10.68 (5.9 to 15.87) | 1.26 (0.61 to 2.01) | -3.02% (-4.03 to -2) |  | 10.22 (5.6 to 15.12) | 157.82 (80.08 to 237.96) | 10.68 (5.9 to 15.87) | 111.95 (54.36 to 177.61) | -3.01% (-4 to -2) |
| Timor-Leste | 11.56 (5.68 to 17.98) | 21.51 (9.45 to 36.49) | 10.86 (5.14 to 17.19) | 13.7 (5.34 to 23.81) | -4.76% (-5.15 to -4.37) |  | 11.56 (5.68 to 17.98) | 1,893.62 (835.97 to 3,212.49) | 10.86 (5.14 to 17.19) | 1,206.96 (471.23 to 2,092.53) | -4.76% (-5.15 to -4.36) |
| Togo | 5.09 (2.93 to 7.34) | 10.59 (5.29 to 17.52) | 5.06 (2.91 to 7.39) | 6.29 (3.17 to 11.02) | -5.65% (-5.87 to -5.43) |  | 5.09 (2.93 to 7.34) | 929.6 (465.67 to 1,533.76) | 5.06 (2.91 to 7.38) | 552.46 (278.92 to 972.1) | -5.64% (-5.85 to -5.42) |
| Tokelau | 11.55 (6.29 to 17.44) | 4.43 (2.17 to 7.24) | 11.39 (6.17 to 17.01) | 2.3 (1.1 to 3.85) | -7.19% (-7.66 to -6.72) |  | 11.55 (6.29 to 17.45) | 388.76 (190.31 to 634.77) | 11.4 (6.17 to 17.02) | 202.14 (96.7 to 339.3) | -7.18% (-7.65 to -6.7) |
| Tonga | 11.95 (6.57 to 17.72) | 4.96 (2.32 to 8.03) | 11.87 (6.58 to 17.53) | 3.24 (1.52 to 5.39) | -4.53% (-4.74 to -4.33) |  | 11.95 (6.57 to 17.73) | 436.44 (204.42 to 706.04) | 11.87 (6.58 to 17.54) | 285.09 (133.76 to 475.48) | -4.52% (-4.73 to -4.31) |
| Trinidad and Tobago | 8.85 (4.73 to 13.35) | 1.81 (0.91 to 2.88) | 8.85 (4.69 to 13.3) | 1.29 (0.61 to 2.21) | -3.63% (-3.96 to -3.31) |  | 8.85 (4.73 to 13.35) | 160.53 (80.3 to 254.57) | 8.85 (4.69 to 13.3) | 113.94 (54.11 to 195.21) | -3.63% (-3.95 to -3.31) |
| Tunisia | 16.95 (11.01 to 23) | 4.14 (2.41 to 6.36) | 16.27 (10.55 to 22.28) | 1.73 (0.98 to 2.78) | -9.43% (-9.7 to -9.15) |  | 16.95 (11.01 to 22.99) | 366.23 (213.41 to 560.84) | 16.28 (10.55 to 22.28) | 153.95 (87.13 to 245.79) | -9.37% (-9.65 to -9.09) |
| Turkey | 19.19 (12.4 to 25.94) | 4.31 (2.53 to 6.73) | 18.26 (11.69 to 24.85) | 2.22 (1.27 to 3.43) | -6.51% (-7.12 to -5.89) |  | 19.19 (12.4 to 25.95) | 379.52 (222.42 to 592.15) | 18.26 (11.68 to 24.85) | 196.46 (112.94 to 301.56) | -6.47% (-7.08 to -5.86) |
| Turkmenistan | 19.27 (12.69 to 26.13) | 55.99 (35.76 to 78.64) | 20.72 (14 to 27.61) | 32.9 (20.24 to 47.68) | -5.81% (-5.96 to -5.65) |  | 19.27 (12.68 to 26.13) | 4,922.28 (3,144.44 to 6,912.35) | 20.72 (14 to 27.61) | 2,890.91 (1,779.75 to 4,188.27) | -5.81% (-5.97 to -5.65) |
| Tuvalu | 10.64 (5.42 to 16.4) | 7.52 (3.48 to 12.57) | 10.39 (5.31 to 15.92) | 3.9 (1.74 to 6.82) | -6.8% (-7.12 to -6.48) |  | 10.64 (5.43 to 16.4) | 661.48 (305.95 to 1,102.84) | 10.39 (5.3 to 15.91) | 343.61 (153.68 to 601.49) | -6.79% (-7.11 to -6.47) |
| Uganda | 2.73 (0.95 to 5.22) | 4.49 (1.44 to 9.6) | 3.27 (1.37 to 5.77) | 3.17 (1.17 to 6.35) | -3.4% (-5.45 to -1.3) |  | 2.74 (0.95 to 5.22) | 394.96 (126.65 to 844.28) | 3.27 (1.37 to 5.77) | 278.98 (103.2 to 559.51) | -3.4% (-5.44 to -1.33) |
| Ukraine | 18.64 (11.65 to 25.18) | 1.74 (1.09 to 2.41) | 19.29 (12.31 to 25.78) | 1.56 (0.91 to 2.3) | 0.14% (-2.24 to 2.58) |  | 18.64 (11.66 to 25.19) | 154.45 (96.65 to 213.9) | 19.29 (12.32 to 25.79) | 138.23 (80.83 to 203.96) | 0.16% (-2.2 to 2.58) |
| United Arab Emirates | 13.16 (8.11 to 18.28) | 0.39 (0.21 to 0.63) | 14.85 (9.49 to 20.12) | 0.21 (0.11 to 0.36) | -5.86% (-6.34 to -5.37) |  | 13.16 (8.11 to 18.28) | 35.32 (19.56 to 56.96) | 14.84 (9.48 to 20.09) | 20.28 (11.17 to 33.9) | -5.5% (-5.95 to -5.04) |
| United Kingdom | 13.79 (8.32 to 19.42) | 0.46 (0.28 to 0.66) | 13.6 (8.3 to 19.4) | 0.35 (0.2 to 0.52) | -2.84% (-3.5 to -2.18) |  | 13.79 (8.32 to 19.42) | 41.12 (25.04 to 59.44) | 13.6 (8.3 to 19.4) | 31.21 (18.54 to 46.14) | -2.8% (-3.46 to -2.14) |
| United Republic of Tanzania | 5.16 (2.19 to 8.97) | 15.93 (6.35 to 28.88) | 6.2 (2.96 to 9.87) | 11.3 (4.71 to 20.38) | -3.5% (-4.32 to -2.68) |  | 5.16 (2.19 to 8.97) | 1,398.43 (558.24 to 2,531.27) | 6.2 (2.96 to 9.88) | 992.13 (413.75 to 1,790.12) | -3.5% (-4.31 to -2.68) |
| United States of America | 12.67 (8.14 to 17.32) | 0.41 (0.27 to 0.57) | 12.82 (8.29 to 17.91) | 0.36 (0.23 to 0.51) | -1.21% (-1.51 to -0.92) |  | 12.66 (8.14 to 17.32) | 37.13 (24.16 to 51.43) | 12.82 (8.3 to 17.9) | 32.76 (21.16 to 46.04) | -1.18% (-1.47 to -0.89) |
| United States Virgin Islands | 7.65 (4.68 to 10.88) | 0.43 (0.23 to 0.67) | 7.99 (4.94 to 11.3) | 0.29 (0.15 to 0.5) | -4.24% (-4.78 to -3.69) |  | 7.65 (4.68 to 10.88) | 38.32 (20.93 to 59.84) | 7.99 (4.92 to 11.29) | 26.57 (13.53 to 44.47) | -4.13% (-4.67 to -3.59) |
| Uruguay | 17.23 (10.69 to 23.89) | 3.09 (1.86 to 4.51) | 16.58 (10.39 to 23.12) | 1.72 (0.92 to 2.71) | -6.47% (-7.6 to -5.32) |  | 17.23 (10.69 to 23.89) | 274.01 (165.21 to 399.04) | 16.58 (10.38 to 23.12) | 153.23 (82.09 to 240.75) | -6.44% (-7.56 to -5.31) |
| Uzbekistan | 10.67 (6.36 to 15.2) | 32.08 (18.15 to 47.33) | 9.84 (5.77 to 14.12) | 17.61 (9.46 to 27.55) | -6.49% (-6.63 to -6.34) |  | 10.67 (6.36 to 15.2) | 2,825.91 (1,600.85 to 4,169.96) | 9.84 (5.77 to 14.14) | 1,551.34 (833.05 to 2,427.99) | -6.48% (-6.62 to -6.34) |
| Vanuatu | 6.24 (2.59 to 10.81) | 7.21 (2.71 to 13.18) | 6.61 (2.83 to 11.32) | 5.14 (1.97 to 9.4) | -3.24% (-3.88 to -2.6) |  | 6.24 (2.59 to 10.81) | 634.16 (238.38 to 1,158.47) | 6.61 (2.83 to 11.32) | 452.33 (173.55 to 827.01) | -3.24% (-3.88 to -2.6) |
| Venezuela (Bolivarian Republic of) | 6.31 (2.89 to 10.45) | 2.46 (1.1 to 4.07) | 6.36 (2.93 to 10.21) | 1.87 (0.76 to 3.31) | -3.27% (-3.8 to -2.74) |  | 6.31 (2.9 to 10.44) | 217.47 (97.28 to 358.94) | 6.36 (2.93 to 10.21) | 164.99 (67.07 to 292.57) | -3.27% (-3.8 to -2.74) |
| Viet Nam | 15.94 (9.65 to 22.33) | 10.42 (5.85 to 15.38) | 15.89 (9.6 to 22.2) | 5.39 (2.94 to 8.81) | -7.05% (-7.21 to -6.89) |  | 15.94 (9.65 to 22.34) | 919.05 (516.66 to 1,355.9) | 15.89 (9.6 to 22.2) | 475.74 (259.6 to 778.25) | -7.04% (-7.2 to -6.88) |
| Yemen | 15.73 (10.12 to 21.66) | 16.95 (8.34 to 30.88) | 15.47 (9.9 to 21.21) | 10.01 (4.95 to 17.74) | -5.38% (-5.88 to -4.87) |  | 15.73 (10.12 to 21.67) | 1,493.07 (736.12 to 2,723.6) | 15.47 (9.9 to 21.22) | 883.29 (436.6 to 1,561.01) | -5.37% (-5.87 to -4.86) |
| Zambia | 4.2 (1.14 to 8.35) | 11.59 (2.95 to 24.91) | 4.52 (1.42 to 8.73) | 5.7 (1.6 to 11.94) | -7.86% (-8.17 to -7.55) |  | 4.2 (1.14 to 8.35) | 1,017.56 (259.05 to 2,183.15) | 4.52 (1.42 to 8.73) | 501.17 (141 to 1,051.33) | -7.85% (-8.17 to -7.53) |
| Zimbabwe | 7.61 (4.17 to 11.36) | 19.67 (10.28 to 30.64) | 7.56 (4.19 to 11.31) | 16.43 (7.86 to 26.43) | -2.47% (-2.98 to -1.96) |  | 7.61 (4.16 to 11.36) | 1,737.04 (907.51 to 2,707.03) | 7.56 (4.19 to 11.32) | 1,448 (693.05 to 2,329.9) | -2.49% (-3 to -1.98) |

**Figure S1.** Global DALYs burden of lower respiratory infections attributable to secondhand smoke among children under 5 years of age in 2019.





**Figure S2.** The correlation between socio-demographic index (SDI) and death rate of lower respiratory infections attributable to secondhand smoke among children under 5 years of age. (A) 21 GBD regions and five SDI regions from 2010 – 2019; (B) 204 countries and territories.

**
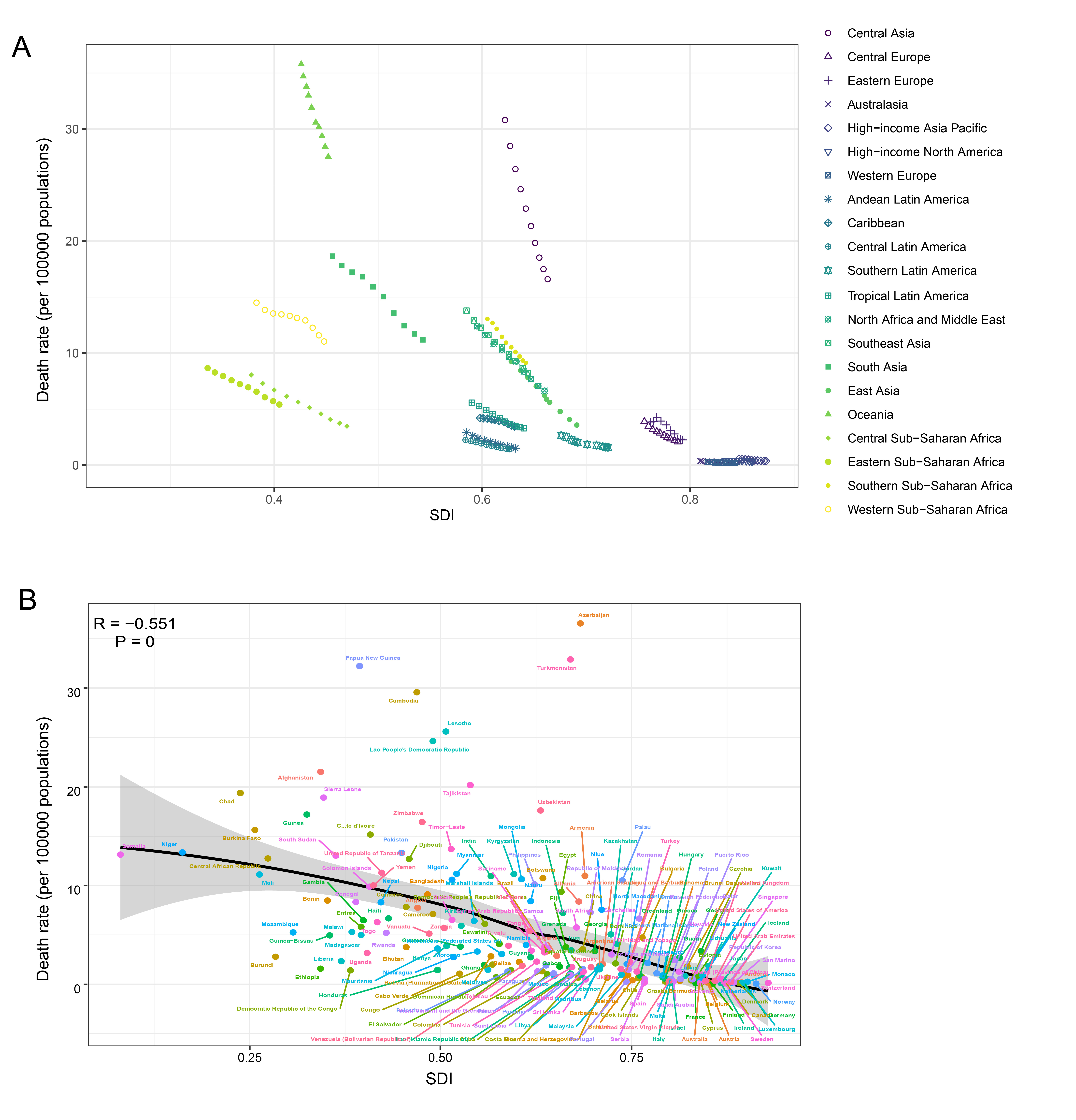
**
